# Disproportionality analysis of adverse event reporting with aspirin, statins, and their co-exposure in FAERS, 2004–2025

**DOI:** 10.3389/fmed.2026.1923188

**Published:** 2026-10-01

**Authors:** Zuofeng Cao, Baojun Xie, Ping Lai, Qijian Wang, Hongzhou Zhang, Bei Wang, Yaxuan Qiu, Qinfu Yan, Jiayuan Ling, Yongling Liao

**Affiliations:** 1 Department of Cardiology, First Affiliated Hospital of Gannan Medical University, Gannan Medical University, Ganzhou, Jiangxi, China; 2 The First School of Clinical Medicine, Gannan Medical University, Ganzhou, Jiangxi, China; 3 Key Laboratory of Prevention and Treatment of Cardiovascular and Cerebrovascular Diseases, Ministry of Education, Gannan Medical University, Ganzhou, China

**Keywords:** aspirin, DDI (Drug-drug interaction), FAERS (FDA adverse eventReporting system), pharmacovigilance, statins

## Abstract

**Objective:**

To characterize and compare adverse-event reporting patterns for aspirin without statins, statins without aspirin, and aspirin–statin co-exposure in the FDA Adverse Event Reporting System (FAERS), and to explore statistical reporting-interaction signals.

**Methods:**

We analyzed FAERS reports from 2004 Q1 through 2025 Q1. Active-comparator disproportionality analyses compared aspirin-statin co-exposure with aspirin-without-statin and statin-without-aspirin reports using the reporting odds ratio (ROR), proportional reporting ratio (PRR), Bayesian confidence propagation neural network (BCPNN), and empirical Bayesian geometric mean (EBGM), followed by Benjamini-Hochberg false discovery rate (BH-FDR) correction. Candidate preferred terms (PTs) common to both active-comparator analyses underwent adjusted statistical interaction analyses using four exposure groups, including a neither-drug reference. Primary models retained missing age, sex, and reporter type as explicit Unknown categories, and a complete-case sensitivity analysis excluded reports with missing adjustment covariates.

**Results:**

After deduplication, 18,932,599 unique FAERS reports were retained. The active-comparator analysis included 134,479 aspirin-without-statin reports, 202,565 statin-without-aspirin reports, and 70,806 aspirin–statin co-exposure reports. Among 9,332 and 9,736 PTs tested in the two active-comparator analyses, 119 and 254 PTs remained after prespecified signal-detection criteria and BH-FDR correction, respectively; 42 PTs were common to both comparisons. Adjusted interaction models were reliably estimable for 40 candidates, and 31 PTs met both additive and multiplicative reporting-interaction criteria. Missingness was substantial for age (42.33%), sex (12.12%), and reporter type (4.35%). The complete-case dataset retained 53.83% of reports, and 21 of the 31 primary both-scale-positive PTs remained jointly positive.

**Conclusions:**

Aspirin-statin co-exposure showed distinct adverse-event reporting patterns in FAERS. The identified signals are exploratory statistical reporting interactions and do not establish increased incidence, clinical risk, causality, or pharmacological drug-drug interactions. Only 21 of the 31 primary both-scale-positive PTs remained jointly positive in the complete-case sensitivity analysis, reinforcing the need for cautious interpretation and validation in denominator-based pharmacoepidemiologic datasets.

## 1 Introduction

Cardiovascular disease (CVD) remains a leading cause of morbidity and mortality worldwide (1). The combination of aspirin and statins serves as a cornerstone therapy in the prevention and management of CVD (2, 3). Aspirin provides antithrombotic benefits through its irreversible inhibition of platelet cyclooxygenase-1 (4), while statins effectively lower low-density lipoprotein cholesterol and stabilize atherosclerotic plaques by inhibiting HMG-CoA reductase (5).This dual-pathway intervention has demonstrated substantial efficacy in reducing major adverse cardiovascular events, solidifying its position in major international guidelines for both secondary and high-risk primary prevention (6). Aspirin and statins are commonly co-prescribed for the prevention and management of CVD.

Regarding their individual safety profiles, aspirin is associated with gastrointestinal bleeding and other hemorrhagic events (7), while statins carry known risks of myotoxicity and hepatotoxicity are well-documented (8). However, the long-term safety of the widely used combination of aspirin and statins, particularly concerning potential adverse drug interactions, warrants rigorous and continuous evaluation. Both drug classes are primarily metabolized via hepatic cytochrome P450 enzymes and can affect liver function and endothelial homeostasis, creating a plausible biological basis for pharmacokinetic and pharmacodynamic interactions (9). The concomitant administration of these drugs may alter the risk landscape through additive effects or synergistic interactions that could unmask or amplify novel toxicities.

Despite widespread co-prescription, large-scale studies designed to distinguish reporting patterns under aspirin-statin co-exposure from those under either drug class alone remain limited. Existing evidence comes mainly from clinical trial subgroup analyses or observational studies focused on efficacy or single outcomes (3, 10). A comprehensive and systematic pharmacovigilance assessment utilizing spontaneous reporting system data which captures real-world, post-marketing safety information is crucial for identifying rare, delayed, or interaction-specific adverse drug reactions (ADRs) that may not be evident in controlled trial settings.

Therefore, this study conducted a disproportionality analysis using FAERS data to compare adverse-event reporting patterns among aspirin-without-statin, statin-without-aspirin, and aspirin–statin co-exposure reports. We further explored adjusted statistical reporting-interaction signals using a neither-drug reference group, with emphasis on prespecified signal detection, multiple-testing control, and cautious non-causal interpretation.

## 2 Methods

### 2.1 Data sources and preprocessing

We conducted a retrospective pharmacovigilance analysis using data extracted from the publicly accessible FDA Adverse Event Reporting System (FAERS; https://open.fda.gov/data/faers/) and followed the READUS-PV reporting principles for disproportionality analyses (11). The query period spanned from the first quarter of 2004 through the first quarter of 2025. Adverse events were analyzed at the Medical Dictionary for Regulatory Activities (MedDRA) Preferred Term (PT) level as recorded in FAERS.

Because spontaneous reporting systems are prone to duplicate entries that can severely distort statistical signals, we implemented a rigorous deduplication pipeline in R (version 4.4.2; R Foundation for Statistical Computing, Vienna, Austria) adhering to FDA-recommended protocols. First, we excluded invalid or withdrawn reports by performing an anti-join between the DEMO table and the FDA's “Deleted.txt” list using the unique CASEID. For patients with multiple submissions reflecting ongoing clinical follow-up (identified by identical CASEIDs), we sorted records chronologically by EVENT_DT or FDA_DT and retained only the most recent entry. In the rare instance of ties sharing both the same CASEID and identical reporting dates, we retained the iteration with the highest PRIMARYID, thereby ensuring that each clinical case was represented uniquely in the final analytic dataset.

### 2.2 Inclusion criteria for target drugs and group classification

We classified target-drug exposure using the standardized DRUGNAME and PROD_AI fields and drug role codes in FAERS. Role codes include Primary Suspect (PS), Secondary Suspect (SS), Concomitant (C), and Interacting (I). Grouping was performed at the unique-report level after deduplication. We prespecified two analytic stages: active-comparator disproportionality analyses restricted to reports in which the target drug was PS or SS, and an adjusted interaction analysis using broader exposure classification with a neither-drug reference group.

#### 2.2.1 Aspirin-without-statin group

For the active-comparator disproportionality analysis, we included reports in which aspirin (including acetylsalicylic acid and relevant synonyms) was listed as PS or SS and the complete medication record contained no statin, regardless of role code (PS, SS, C, or I).

#### 2.2.2 Statin-without-aspirin group

The revised analysis included seven marketed statins: atorvastatin, fluvastatin, lovastatin, pitavastatin, pravastatin, rosuvastatin, and simvastatin, together with relevant synonym and product-ingredient mappings. Reports were eligible if at least one statin was implicated as PS or SS and the complete medication record contained no aspirin, regardless of its role code. Cerivastatin was not included because it was withdrawn before the study period.

#### 2.2.3 Aspirin-statin co-exposure group

For active-comparator analyses, reports were required to list both aspirin and a statin, with at least one of the two target agents designated as PS or SS. For adjusted interaction analyses, exposure was reclassified into four mutually exclusive categories-aspirin without statins, statins without aspirin, aspirin-statin co-exposure, and neither aspirin nor statins, so that the reporting-interaction model had an explicit background comparator.

### 2.3 Disproportionality analysis of adverse events

Disproportionality analysis, also known as case-non-case analysis, is widely used for pharmacovigilance signal detection (12, 13). The reporting odds ratio (ROR), proportional reporting ratio (PRR), Bayesian confidence propagation neural network (BCPNN), and empirical Bayesian geometric mean (EBGM) were used as complementary measures. A PT was considered a candidate signal only when all four prespecified criteria were met and the signal remained after Benjamini–Hochberg false discovery rate (BH-FDR) correction.

For active-comparator analyses, separate 2 × 2 contingency tables were constructed for co-exposure vs. aspirin without statins and for co-exposure vs. statins without aspirin. For each PT, a denotes co-exposure reports containing the target PT; b denotes co-exposure reports not containing the target PT; c denotes comparator reports containing the target PT; and d denotes comparator reports not containing the target PT. N denotes the total number of reports in the corresponding 2 × 2 table (N=a+b+c+d). The corresponding calculation formulas and criteria for positive signal determination are presented below.

The formula for calculating the Reporting Odds Ratio (ROR) is

$$\mathrm{ROR}=\frac{a\times d}{b\times c}.$$

The formula for calculating its 95% Confidence Interval (CI) is

$$\$95\text{\%}\mathrm{CI}=e^{\ln(\mathrm{ROR})\pm 1.96\times \sqrt{\left(\frac{1}{a}\right)+\left(\frac{1}{b}\right)+\left(\frac{1}{c}\right)+\left(\frac{1}{d}\right)}.}\$$$

The positive criterion was defined as: the number of reports for the target event a ≥ 3, and the lower limit of the 95% confidence interval > 1. The formula for calculating the Proportional Reporting Ratio (PRR) is PRR=[a/(a+b)][c/[c/(c+d)]. To verify statistical significance, this analysis is supplemented by a Chi-square test, with the formula:

$$\chi^{2}=\frac{{\left(a\times d-b\times c\right)}^{2}\times (a+b+c+d)}{\left(a+b)\times (c+d)\times (a+c)\times (b+d\right)}$$

The positive criterion was defined as: the number of reports for the target event a ≥ 3, PRR ≥ 2, and *χ*^2^ ≥ 4. The Bayesian Confidence Propagation Neural Network (BCPNN) measures signal association strength by calculating the Information Component (IC), defined by the core formula:

IC=log_2(a(a+b+c+d)/(a+c)(a+b))

The unshrunken observed-to-expected ratio (RRR) is calculated using the formula:

$$\mathrm{RRR}=\frac{a\times (a+b+c+d)}{\left(a+c)\times (a+b\right)}$$

A BCPNN signal was considered positive when IC025 exceeded 0. EBGM and its 5th posterior percentile (EBGM05) were estimated separately using empirical Bayes shrinkage; the raw RRR formula was not interpreted as EBGM. An EBGM signal was considered positive when EBGM05 exceeded 2. A PT was retained as a candidate signal only when it met the ROR, PRR, BCPNN, and EBGM criteria and remained after BH-FDR correction within the corresponding active-comparator testing set.

### 2.4 Statistical reporting-interaction models

Because FAERS lacks exposed-population denominators, true incidence rates, risk differences, and relative risks cannot be estimated. We therefore evaluated statistical reporting interaction within a case–non-case framework. These analyses assess whether co-reporting patterns depart from the specified interaction null; they do not establish a pharmacological drug–drug interaction or causal clinical effect (13).

For each of the 42 candidate PTs common to the two active-comparator analyses, we constructed a multivariable logistic regression model:

$$\mathrm{Logit}(P)=\beta_{0}+\beta_{1}A+\beta_{2}B+\beta_{3}(A\times B)+\sum i\gamma \mathrm{iCi}$$

In this model, A and B indicate aspirin and statin exposure, respectively, and the neither-drug category served as the reference. Ci denotes prespecified covariates, including age group, sex, reporting year, and reporter type. The model outcome was whether the target PT was recorded in the report.

Exp(*β*_1_) represents the adjusted reporting odds for aspirin without statins relative to the neither-drug reference;
Exp(*β*_2_) represents the adjusted reporting odds for statins without aspirin relative to the neither-drug reference;
Exp(*β*_1_ + *β*_2_ + *β*_3_) represents the adjusted reporting odds for aspirin–statin co-exposure relative to the neither-drug reference;
Exp(*β*_3_) yields the multiplicative interaction ratio (IR), indicating whether the reporting odds for co-exposure depart from the product of the corresponding single-exposure reporting odds. A positive multiplicative reporting interaction was defined as an IR greater than 1 with statistical support after the prespecified multiple-testing procedure.

To evaluate interaction on an additive scale using odds-ratio-based estimates, we calculated the OR-based relative excess due to interaction:

$$\mathrm{RERI}=OR_{\mathrm{AB}}-OR_{A}-OR_{B}+1$$

where OR_AB_ = exp (*β*_1_ + *β*_2_ + *β*_3_), OR_A_ = exp (*β*_1_), and OR_B_ = exp (*β*_2_). A positive OR-based RERI with a 95% confidence interval excluding zero was considered a positive additive reporting interaction. This OR-scale measure does not represent an absolute excess risk. Confidence intervals were estimated using the delta method for IR and bootstrap resampling for OR-based RERI.

All statistical analyses were performed in R 4.4.2. Sparse-data stability was assessed through the four-cell counts, width of confidence intervals, and a complete-case sensitivity analysis. Missing covariate data were summarized by exposure group, and estimates based on sparse or highly missing data were interpreted cautiously.

Missing-data handling and robustness were prespecified as important sensitivity considerations. Missing age, sex, and reporter type were retained as explicit Unknown categories in the primary models; reporting year was complete. The complete-case sensitivity analysis excluded reports with missing adjustment covariates.

## 3 Results

### 3.1 Data overview and spectrum of adverse events

After downloading 22,775,812 original DEMO records and performing deduplication and data cleaning, 18,932,599 unique FAERS reports were retained (Figure 1). The active-comparator analysis included 134,479 aspirin-without-statin reports, 202,565 statin-without-aspirin reports, and 70,806 aspirin-statin co-exposure reports. Baseline characteristics, including sex, age, serious outcomes, reporting year, and reporting country, are summarized in Table 1.

**Figure 1:**
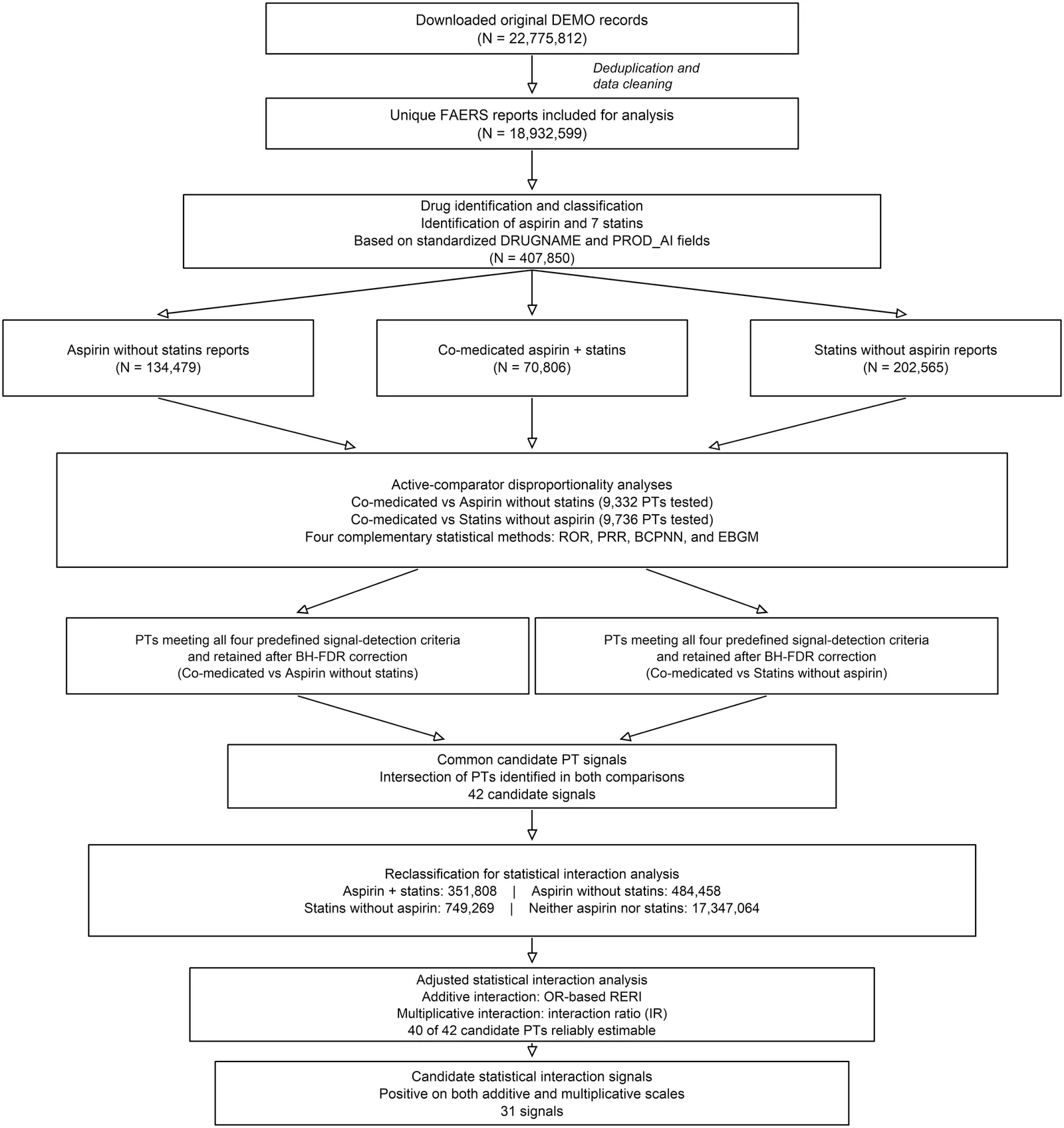
Data screening and analysis workflow.

**Table 1:** Characteristics of FAERS reports by exposure group.

Category	Sub_category	Aspirin without statins	Statins without aspirin	Co-medicated
Gender	Female	48,360 (35.96%)	98,052 (48.41%)	27,306 (38.56%)
	Male	45,083 (33.52%)	82,702 (40.83%)	39,214 (55.38%)
	Unknown or missing	41,036 (30.51%)	21,811 (10.77%)	4,286 (6.05%)
Age (years)	<18	2,759 (2.05%)	607 (0.30%)	133 (0.19%)
	18–64	29,062 (21.61%)	63,477 (31.34%)	18,977 (26.80%)
	65–84	36,975 (27.49%)	62,988 (31.10%)	34,052 (48.09%)
	≥85	7,921 (5.89%)	6,894 (3.40%)	4,451 (6.29%)
	Unknown or missing	57,748 (42.94%)	68,473 (33.80%)	13,130 (18.54%)
Serious outcome	OT	53,926 (40.10%)	101,921 (50.32%)	34,461 (48.67%)
	CA	1,516 (1.13%)	508 (0.25%)	246 (0.35%)
	HO	51,801 (38.52%)	47,751 (23.57%)	35,757 (50.50%)
	RI	690 (0.51%)	2,305 (1.14%)	295 (0.42%)
	DS	1,358 (1.01%)	11,555 (5.70%)	3,483 (4.92%)
	LT	6,529 (4.86%)	8,314 (4.10%)	5,378 (7.60%)
	DE	34,930 (25.97%)	12,894 (6.37%)	5,873 (8.29%)
Report Year	2004	1,862 (1.38%)	5,712 (2.82%)	1,618 (2.29%)
	2005	2,132 (1.59%)	5,490 (2.71%)	1,831 (2.59%)
	2006	1,206 (0.90%)	5,719 (2.82%)	1,642 (2.32%)
	2007	1,390 (1.03%)	4,906 (2.42%)	1,377 (1.94%)
	2008	2,485 (1.85%)	7,549 (3.73%)	2,449 (3.46%)
	2009	2,233 (1.66%)	8,519 (4.21%)	3,162 (4.47%)
	2010	2,146 (1.60%)	13,530 (6.68%)	3,554 (5.02%)
	2011	2,633 (1.96%)	10,705 (5.28%)	2,573 (3.63%)
	2012	4,150 (3.09%)	13,834 (6.83%)	3,332 (4.71%)
	2013	4,170 (3.10%)	10,506 (5.19%)	3,150 (4.45%)
	2014	5,230 (3.89%)	13,954 (6.89%)	3,152 (4.45%)
	2015	6,155 (4.58%)	10,374 (5.12%)	3,978 (5.62%)
	2016	10,291 (7.65%)	10,234 (5.05%)	4,104 (5.80%)
	2017	11,243 (8.36%)	8,043 (3.97%)	3,322 (4.69%)
	2018	31,540 (23.45%)	9,893 (4.88%)	5,468 (7.72%)
	2019	8,184 (6.09%)	12,172 (6.01%)	5,097 (7.20%)
	2020	12,292 (9.14%)	10,996 (5.43%)	5,538 (7.82%)
	2021	6,794 (5.05%)	8,546 (4.22%)	3,934 (5.56%)
	2022	4,685 (3.48%)	8,618 (4.25%)	3,578 (5.05%)
	2023	5,985 (4.45%)	9,935 (4.90%)	3,268 (4.62%)
	2024	5,936 (4.41%)	10,497 (5.18%)	3,581 (5.06%)
	2025	1,737 (1.29%)	2,833 (1.40%)	1,098 (1.55%)
Reported Countries (Top five)	United States of America	87,590 (67.66%)	104,651 (54.65%)	24,992 (37.38%)
	France, French Republic	6,086 (4.70%)	13,522 (7.06%)	8,289 (12.40%)
	United Kingdom of Great Britain & Northern Ireland	NA	19,952 (10.42%)	7,248 (10.84%)
	Germany, Federal Republic of	4,082 (3.15%)	5,761 (3.01%)	4,671 (6.99%)
	Canada		8,908 (4.65%)	4,366 (6.53%)
	Japan	4,665 (3.60%)	NA	NA
	Italy, Italian Republic	4,261 (3.29%)	NA	NA

Values are presented as *n* (%) unless otherwise specified. Percentages are calculated using all reports within each exposure group as the denominator. Continuous age was compared using the Kruskal–Wallis test. Sex and reporter-type comparisons used reports with available information; unknown/other categories were retained for descriptive presentation. Categorical comparisons used Pearson's chi-square test or Fisher's exact test, as appropriate. Outcomes were not mutually exclusive and were analyzed separately as binary variables. OT, other serious outcome; CA, congenital anomaly; HO, hospitalization; RI, required intervention; DS, disability; LT, life-threatening; DE, death; NA, no available.

In the active-comparator disproportionality analyses, 9,332 PTs were tested for co-exposure vs. aspirin without statins and 9,736 PTs for co-exposure vs. statins without aspirin (Table 2). After applying all four prespecified signal-detection criteria and BH-FDR correction, 119 and 254 PTs remained in the two analyses, respectively. Forty-two PTs were common to both BH-FDR-positive sets and were carried forward to the adjusted statistical interaction analysis (Figure 2). Full results are presented in Supplementary Tables S1, S2, and side-by-side results for the 42 common candidates are presented in Supplementary Table S3.

**Table 2:** Summary of the two active-comparator disproportionality analyses.

Item	Co-medicated vs. Aspirin without statins	Co-medicated vs. Statins without aspirin
Co-medicated reports	70,806	70,806
Comparator reports	134,479	202,565
PTs tested	9,332	9,736
ROR positive	2,002	1,936
PRR positive	2,123	1,661
BCPNN positive	1,432	1,391
EBGM positive	119	254
BH-FDR-positive PTs	119	254

Forty-two PTs were common to the two BH-FDR-positive sets. BH-FDR correction was applied separately across all 9,332 and 9,736 tested PTs in the respective active-comparator analyses.

**Figure 2:**
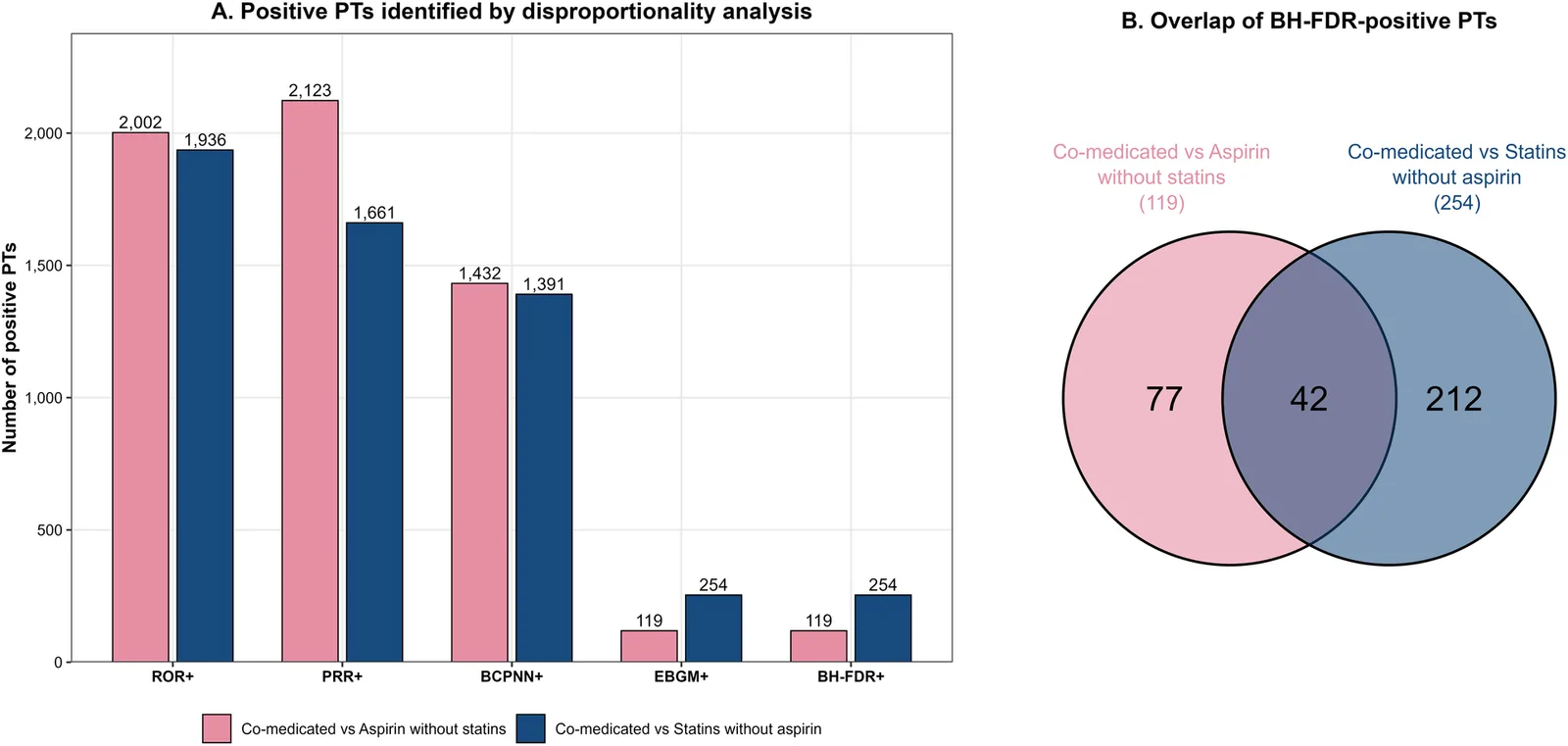
Active-comparator disproportionality analyses. **(A)** Number of PTs meeting the ROR, PRR, BCPNN, EBGM, and BH-FDR criteria for aspirin-statin co-exposure vs. aspirin without statins and versus statins without aspirin. **(B)** Overlap of BH-FDR-positive PTs between the two active-comparator analyses; 42 PTs were common to both sets.

### 3.2 Distribution by system organ class (SOC)

The 42 common candidate PTs were distributed across multiple primary SOCs, most frequently nervous system disorders (7 PTs), respiratory, thoracic and mediastinal disorders (4 PTs), investigations (4 PTs), gastrointestinal disorders (4 PTs), reproductive system and breast disorders (3 PTs), psychiatric disorders (3 PTs), injury, poisoning and procedural complications (3 PTs), and congenital, familial and genetic disorders (3 PTs) (Figure 3). This distribution describes the candidate reporting signals and does not imply multi-organ clinical toxicity.

**Figure 3:**
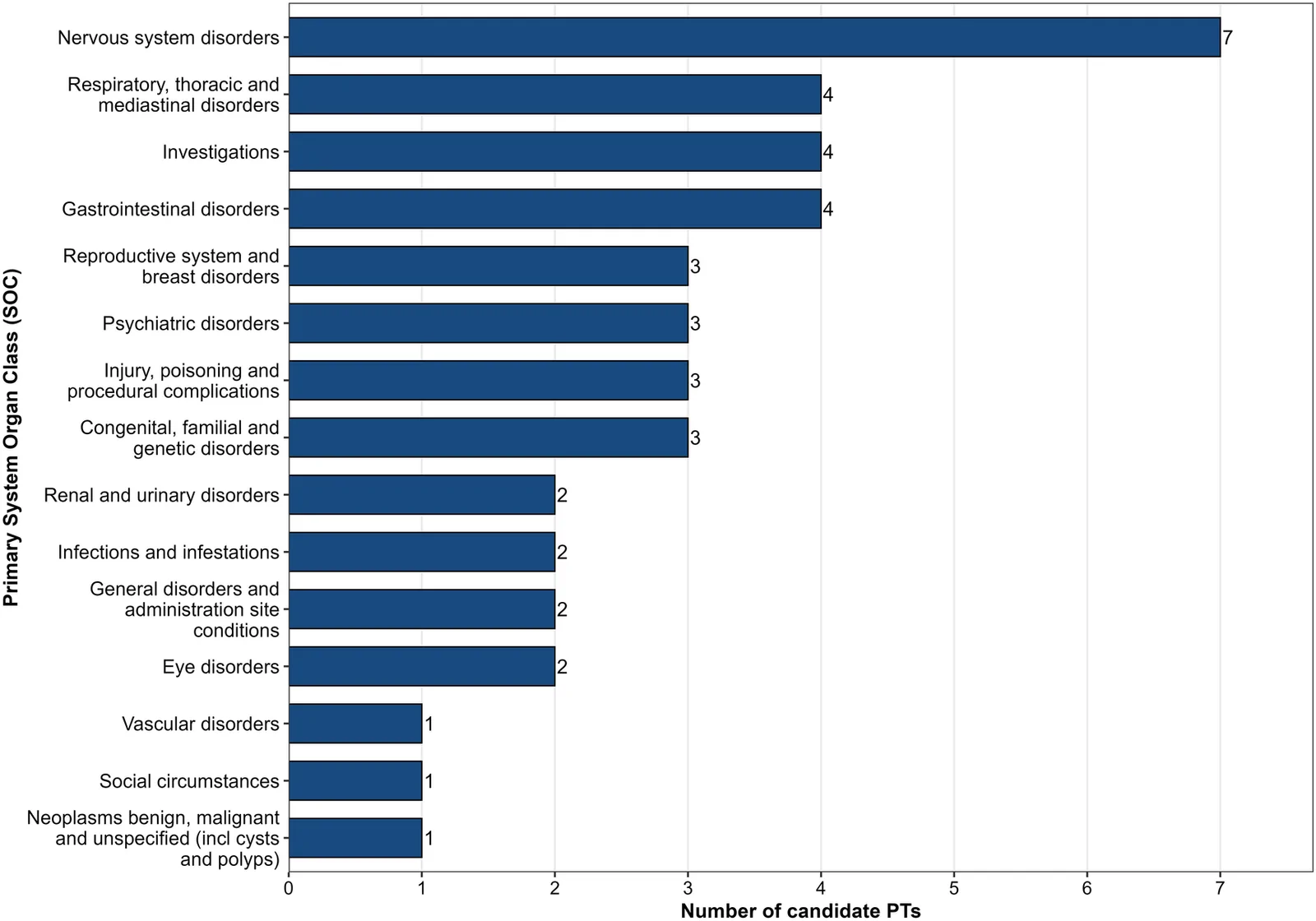
Distribution of the 42 common candidate PTs across primary system organ classes (SOCs). The counts describe candidate reporting signals identified in both active-comparator analyses and do not represent the number of affected patients or confirmed clinical adverse reactions.

### 3.3 Empirical Bayes estimates Among candidate PTs

Figure 4 presents EBGM estimates for the 42 common candidate PTs in the two active-comparator analyses. These estimates quantify disproportionate reporting relative to the active comparators and are hypothesis-generating measures rather than incidence risks. Candidate PTs with sparse counts, wide intervals, non-specific terms, or plausible coding and reporting artifacts require cautious interpretation and manual clinical review.

**Figure 4:**
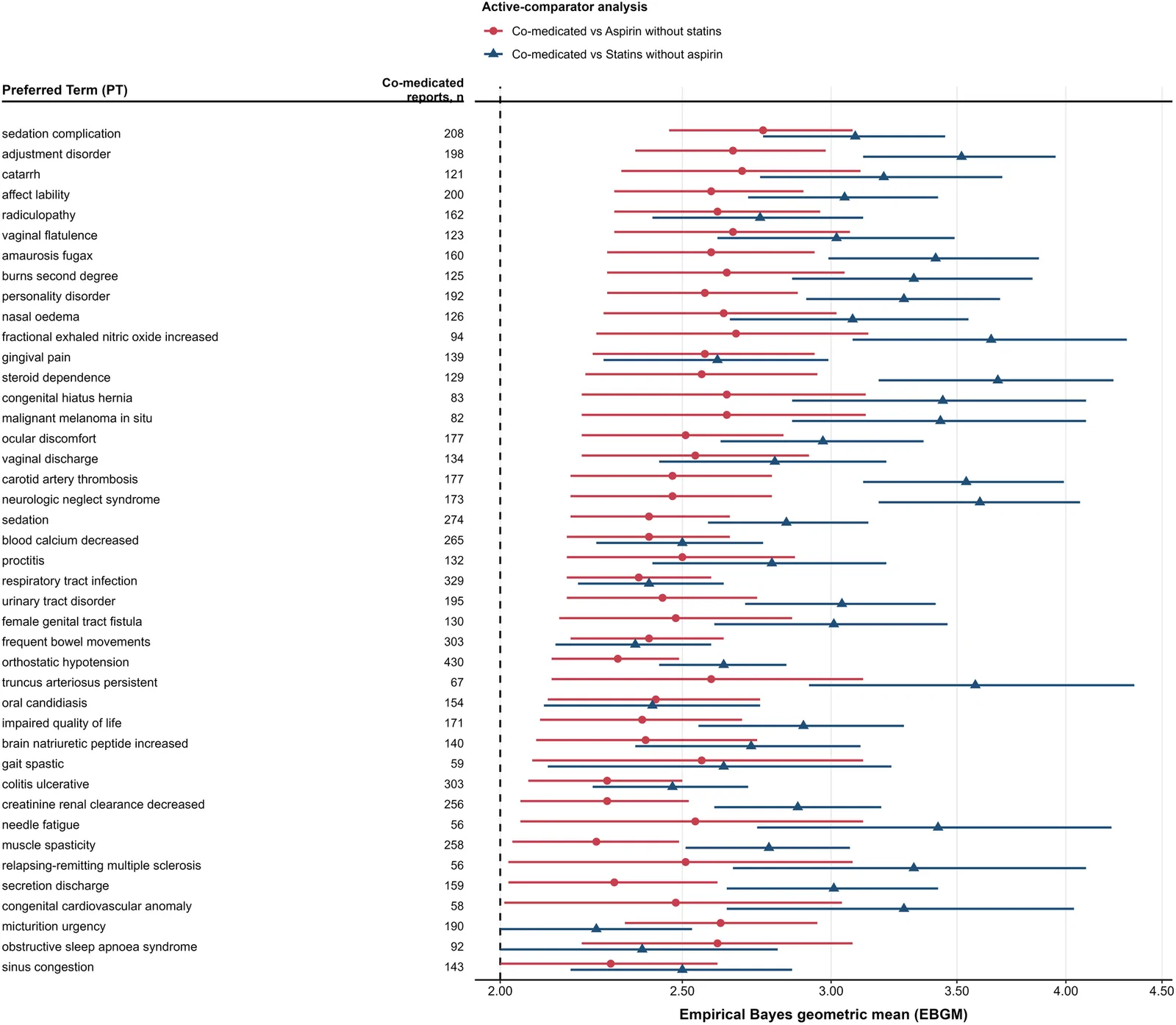
Empirical Bayes geometric mean (EBGM) estimates for the 42 common candidate PTs in the two active-comparator analyses. Points and intervals show disproportionate reporting estimates for aspirin-statin co-exposure versus aspirin without statins and versus statins without aspirin. These estimates quantify reporting associations and do not represent incidence risks.

### 3.4 Adjusted statistical reporting-interaction analysis

For the interaction analysis, reports were reclassified into four mutually exclusive exposure groups: 484,458 aspirin-without-statin reports, 749,269 statin-without-aspirin reports, 351,808 aspirin-statin co-exposure reports, and 17,347,064 reports listing neither drug class. Forty of the 42 candidate PTs were reliably estimable in the adjusted models. Thirty-one PTs met both the additive and multiplicative reporting-interaction criteria and are listed in Table 3. These findings represent statistical pharmacovigilance interaction signals only.

**Table 3:** Candidate PTs with positive adjusted statistical interactions on both additive and multiplicative scales.

PT	Primary SOC	PT reports, *n*	OR-based RERI (95% CI)	IR-based RERI (95% CI)
Muscle spasticity	Nervous system disorders	11,603	1.56 (1.30–1.82)	3.20 (2.62–3.90)
sedation	Nervous system disorders	21,882	0.79 (0.65–0.94)	2.29 (1.97–2.65)
Affect lability	Psychiatric disorders	8,974	1.75 (1.43–2.07)	3.81 (3.00–4.85)
Colitis ulcerative	Gastrointestinal disorders	37,949	0.64 (0.51–0.76)	1.96 (1.72–2.22)
Vaginal discharge	Reproductive system and breast disorders	9,327	1.38 (1.10–1.67)	4.07 (3.10–5.35)
Congenital cardiovascular anomaly	Congenital, familial and genetic disorders	798	10.79 (8.00–13.57)	282.46 (84.58–943.28)
Adjustment disorder	Psychiatric disorders	1,123	14.64 (12.03–17.25)	5.83 (3.73–9.11)
Burns second degree	Injury, poisoning and procedural complications	1,625	5.08 (3.95–6.21)	5.46 (3.48–8.55)
Personality disorder	Psychiatric disorders	2,364	4.75 (3.76–5.75)	3.25 (2.34–4.51)
Steroid dependence	General disorders and administration site conditions	628	25.89 (20.48–31.31)	42.61 (14.89–121.91)
Sedation complication	Injury, poisoning and procedural complications	1,866	6.59 (5.28–7.90)	3.76 (2.52–5.60)
Female genital tract fistula	Reproductive system and breast disorders	1,492	5.33 (3.99–6.68)	3.46 (2.28–5.26)
Nasal oedema	Respiratory, thoracic and mediastinal disorders	1,584	4.33 (3.20–5.46)	3.10 (2.09–4.60)
Catarrh	Respiratory, thoracic and mediastinal disorders	785	12.14 (9.33–14.95)	9.91 (4.42–22.24)
Urinary tract disorder	Renal and urinary disorders	4,027	2.21 (1.65–2.76)	1.98 (1.55–2.54)
Relapsing-remitting multiple sclerosis	Nervous system disorders	762	6.57 (4.52–8.62)	7.84 (3.72–16.51)
Needle fatigue	Injury, poisoning and procedural complications	388	17.28 (12.27–22.28)	50.42 (10.44–243.47)
Impaired quality of life	Social circumstances	12,824	0.63 (0.32–0.95)	1.55 (1.28–1.86)
Frequent bowel movements	Gastrointestinal disorders	23,452	0.48 (0.30–0.66)	1.33 (1.17–1.51)
Proctitis	Gastrointestinal disorders	2,480	2.31 (1.58–3.04)	2.02 (1.48–2.77)
Carotid artery thrombosis	Nervous system disorders	689	19.87 (15.40–24.35)	2.56 (1.62–4.03)
Truncus arteriosus persistent	Congenital, familial and genetic disorders	171	167.05 (106.80–227.30)	74.25 (8.73–631.38)
Amaurosis fugax	Eye disorders	884	11.98 (9.17–14.78)	2.16 (1.43–3.27)
Malignant melanoma *in situ*	Neoplasms benign, malignant and unspecified (incl cysts and polyps)	1,250	2.58 (1.59–3.56)	1.91 (1.31–2.78)
Ocular discomfort	Eye disorders	8,852	0.88 (0.51–1.24)	1.39 (1.13–1.69)
Neurologic neglect syndrome	Nervous system disorders	418	54.69 (41.22–68.16)	2.39 (1.35–4.21)
Radiculopathy	Nervous system disorders	2,434	4.21 (3.13–5.29)	1.44 (1.10–1.87)
Creatinine renal clearance decreased	Investigations	3,586	1.61 (1.09–2.13)	1.32 (1.08–1.62)
Blood calcium decreased	Investigations	10,226	0.83 (0.53–1.13)	1.22 (1.05–1.41)
Vaginal flatulence	Reproductive system and breast disorders	243	78.11 (53.76–102.46)	4.73 (1.40–15.97)
Gingival pain	Gastrointestinal disorders	7,292	0.64 (0.31–0.98)	1.27 (1.04–1.53)

All PTs shown met the prespecified criteria for positive interaction on both additive and multiplicative scales, including BH-FDR < 0.05. Exact BH-FDR values are provided in the Supplementary Table.

PT reports, *n* represents the number of unique FAERS reports containing the corresponding PT across the four exposure groups included in the statistical interaction analysis.

IR, interaction ratio on the multiplicative scale; OR-based RERI, relative excess due to interaction on the odds scale; BH-FDR, Benjamini-Hochberg false discovery rate.

These findings represent statistical pharmacovigilance interaction signals and do not establish causal pharmacological drug-drug interactions.

Missing-data and complete-case sensitivity analyses showed that age was missing or unknown for 8,013,417 reports (42.33%), sex for 2,295,134 reports (12.12%), and reporter type for 823,543 reports (4.35%); reporting year was complete. The complete-case dataset retained 10,190,569 of 18,932,599 reports (53.83%). Among the 31 primary both-scale-positive PTs, 21 remained jointly positive in the complete-case analysis, 6 remained directionally consistent but no longer jointly significant, 3 were not fully directionally consistent, and 1 was not reliably estimable (Supplementary Tables S5, S6).

## 4 Discussion

In this updated FAERS analysis, aspirin-without-statin, statin-without-aspirin, and aspirin-statin co-exposure reports showed distinct reporting patterns. The revised active-comparator workflow applied four complementary disproportionality algorithms and BH-FDR correction, yielding 119 and 254 retained PTs in the two comparisons and 42 common candidate PTs. Forty candidates were reliably estimable in adjusted four-group interaction models, of which 31 met both additive and multiplicative reporting-interaction criteria. Importantly, these results identify statistical reporting interactions rather than established pharmacological DDIs.

Established safety profiles remain central to interpretation: aspirin is most consistently associated with gastrointestinal and bleeding events, whereas statins are associated with musculoskeletal symptoms and laboratory abnormalities. Severe statin myopathy is uncommon in clinical practice, and symptom reporting may be influenced by nocebo effects, although modest risks of creatine kinase elevation and dysglycemia persist (14). For aspirin, increased gastrointestinal bleeding and dyspepsia remain the principal concerns in routine care (15).

Compared with either drug class without the other, the broader candidate-signal distribution observed under co-exposure may reflect baseline cardiovascular risk, older age, comorbidity burden, polypharmacy, healthcare contact, diagnostic testing, and differential reporting intensity (16). The predominance of signals within the MedDRA System Organ Class (SOC) investigations is consistent with enhanced surveillance among patients receiving long-term cardioprotective regimens (17). Apparent respiratory and ophthalmic drug–drug interaction signals warrant focused evaluation but should be interpreted cautiously given potential confounding by indication and differential monitoring intensity.

With respect to ophthalmic events such as amaurosis fugax, there is no established mechanism supporting a direct aspirin–statin interaction that would cause transient monocular vision loss (18). A more plausible explanation is confounding by indication: patients with carotid atherosclerosis or thromboembolic risk are more likely to receive combined aspirin–statin therapy and inherently have higher rates of transient ischemic ocular events (19, 20). Rigorous validation with imaging and careful temporal anchoring of exposure and outcomes is needed before inferring causality.

For respiratory events, including wheeze, exertional dyspnea, and abnormal breath sounds, aspirin can exacerbate respiratory disease in susceptible individuals (aspirin-exacerbated respiratory disease, AERD) (21). Statins have an inconsistent respiratory safety signal, with rare case reports of interstitial lung disease and potential indirect effects via myopathy-related respiratory muscle weakness. The observed interaction may therefore largely reflect AERD phenotypes in a subset of patients, with statin use serving as a marker of cardiovascular risk and healthcare contact rather than indicating a true pharmacodynamic interaction.

For neurologic and reproductive-system candidate PTs, including radiculopathy, neurologic neglect syndrome, vaginal discharge, and vaginal flatulence, alternative explanations such as age, diabetes, cerebrovascular disease, pelvic or gastrointestinal conditions, coding artifacts, and reporting clusters should be considered. No causal mechanism for an aspirin-statin interaction should be inferred from the present results.

Complete-case sensitivity analysis further tempered interpretation of the less robust candidate signals. Amaurosis fugax, ocular discomfort, and impaired quality of life were not fully consistent across analyses; congenital cardiovascular anomaly was not reliably estimable in the complete-case dataset; and truncus arteriosus persistent and vaginal flatulence showed substantial instability or loss of joint statistical significance. These signals should therefore not be emphasized as robust clinical findings without case-level review and external validation. Future sensitivity analyses should examine healthcare-professional reports, multiple-imputation approaches, statin subtypes, calendar-time strata, and clinically prespecified outcome groups.

## 5 Limitations

Several limitations warrant emphasis. First, FAERS lacks exposed-population denominators; therefore, incidence, absolute risk, risk difference, and relative risk cannot be estimated. Second, spontaneous reports are subject to underreporting, selective reporting, duplicate records, coding variability, and variable report quality. Third, information on comorbidities, indication severity, dose, duration, time to onset, dechallenge, and rechallenge is incomplete. Fourth, missingness was substantial: age was missing or unknown for 42.33% of reports, sex for 12.12%, and reporter type for 4.35%. The primary models retained these values as Unknown categories, whereas the complete-case analysis retained only 53.83% of reports. Fifth, only 21 of the 31 primary both-scale-positive PTs remained jointly positive in the complete-case analysis; the others lost joint significance, showed incomplete directional consistency, or were not estimable. Sixth, residual confounding by indication, comorbidity, concomitant medications, healthcare utilization, and notoriety effects cannot be excluded. Finally, sparse cells and large-scale multiplicity can produce unstable or false-positive signals despite algorithm intersection and BH-FDR correction, and the interaction estimates represent reporting interactions only.

## 6 Conclusions

In FAERS, aspirin-statin co-exposure was associated with distinct adverse-event reporting patterns compared with either drug class without the other. Established aspirin-related bleeding and statin-related muscle and liver concerns remain the most clinically interpretable findings. The additional candidate PTs identified through active-comparator and interaction analyses are exploratory reporting signals rather than evidence of increased incidence, clinical risk, causality, or pharmacological drug-drug interactions. Only 21 of the 31 primary both-scale-positive PTs remained jointly positive in the complete-case sensitivity analysis, further supporting cautious interpretation. These findings warrant clinical review and validation in denominator-based pharmacoepidemiologic studies.

## Data Availability

Publicly available datasets were analyzed in this study. This data can be found here: Repository Name: FDA Adverse Event Reporting System (FAERS) Quarterly Data FilesDirect Link to Data: https://open.fda.gov/ data/faers/.
