# Supplementary material for: Disproportionality analysis of adverse event reporting with aspirin, statins, and their co-exposure in FAERS, 2004–2025
Source: Front Med (Lausanne). 2026 Oct 1;13:1923188. doi: 10.3389/fmed.2026.1923188 (PMC13669111; doi:10.3389/fmed.2026.1923188)
Supplement: Supplementary file 1 [file Table1.docx]

| PT | Primary SOC | a | b | c | d | ROR (95% CI) | PRR (95% CI) | Chi-square | IC (IC025) | EBGM (EB05-EB95) | Fisher P | BH-FDR |
| --- | --- | --- | --- | --- | --- | --- | --- | --- | --- | --- | --- | --- |
| adjustment disorder | Psychiatric disorders | 198 | 70,608 | 10 | 134,469 | 37.71 (19.98-71.18) | 37.61 (19.92-70.98) | 339.53 | 1.44 (1.21) | 2.66 (2.36-2.98) | <0.001 | <0.001 |
| affect lability | Psychiatric disorders | 200 | 70,606 | 16 | 134,463 | 23.81 (14.30-39.61) | 23.74 (14.27-39.50) | 323.05 | 1.41 (1.17) | 2.59 (2.30-2.90) | <0.001 | <0.001 |
| alanine aminotransferase increased | Investigations | 873 | 69,933 | 312 | 134,167 | 5.37 (4.72-6.11) | 5.31 (4.67-6.05) | 809.72 | 1.09 (0.99) | 2.12 (2.01-2.24) | <0.001 | <0.001 |
| amaurosis fugax | Eye disorders | 160 | 70,646 | 11 | 134,468 | 27.69 (15.03-51.01) | 27.63 (15.00-50.89) | 264.34 | 1.42 (1.16) | 2.59 (2.28-2.94) | <0.001 | <0.001 |
| arterial occlusive disease | Vascular disorders | 328 | 70,478 | 60 | 134,419 | 10.43 (7.92-13.73) | 10.38 (7.89-13.67) | 430.88 | 1.28 (1.11) | 2.40 (2.20-2.63) | <0.001 | <0.001 |
| arthralgia | Musculoskeletal and connective tissue disorders | 2,192 | 68,614 | 750 | 133,729 | 5.70 (5.24-6.19) | 5.55 (5.11-6.03) | 2115.24 | 1.11 (1.04) | 2.16 (2.08-2.23) | <0.001 | <0.001 |
| balance disorder | Nervous system disorders | 771 | 70,035 | 266 | 134,213 | 5.55 (4.83-6.39) | 5.50 (4.79-6.33) | 732.81 | 1.10 (0.99) | 2.14 (2.02-2.27) | <0.001 | <0.001 |
| blood calcium decreased | Investigations | 265 | 70,541 | 47 | 134,432 | 10.75 (7.88-14.66) | 10.71 (7.85-14.60) | 351.91 | 1.29 (1.09) | 2.40 (2.17-2.65) | <0.001 | <0.001 |
| blood cholesterol abnormal | Investigations | 154 | 70,652 | 8 | 134,471 | 36.64 (18.00-74.58) | 36.56 (17.96-74.41) | 263.25 | 1.44 (1.17) | 2.62 (2.30-2.98) | <0.001 | <0.001 |
| blood cholesterol increased | Investigations | 911 | 69,895 | 156 | 134,323 | 11.22 (9.47-13.30) | 11.09 (9.36-13.14) | 1229.27 | 1.30 (1.20) | 2.46 (2.33-2.59) | <0.001 | <0.001 |
| blood creatine phosphokinase increased | Investigations | 1,578 | 69,228 | 121 | 134,358 | 25.31 (21.03-30.46) | 24.77 (20.59-29.79) | 2584.75 | 1.43 (1.34) | 2.68 (2.57-2.79) | <0.001 | <0.001 |
| blood glucose increased | Investigations | 1,064 | 69,742 | 253 | 134,226 | 8.09 (7.05-9.29) | 7.99 (6.97-9.16) | 1257.47 | 1.22 (1.13) | 2.33 (2.21-2.45) | <0.001 | <0.001 |
| blood triglycerides increased | Investigations | 385 | 70,421 | 22 | 134,457 | 33.41 (21.74-51.35) | 33.24 (21.63-51.07) | 651.99 | 1.45 (1.28) | 2.69 (2.47-2.92) | <0.001 | <0.001 |
| body height decreased | Investigations | 203 | 70,603 | 31 | 134,448 | 12.47 (8.54-18.20) | 12.44 (8.52-18.15) | 283.17 | 1.31 (1.09) | 2.44 (2.17-2.73) | <0.001 | <0.001 |
| brain natriuretic peptide increased | Investigations | 140 | 70,666 | 22 | 134,457 | 12.11 (7.72-18.98) | 12.09 (7.71-18.94) | 193.49 | 1.30 (1.03) | 2.39 (2.09-2.74) | <0.001 | <0.001 |
| burns second degree | Injury, poisoning and procedural complications | 125 | 70,681 | 4 | 134,475 | 59.46 (21.97-160.91) | 59.35 (21.93-160.61) | 222.50 | 1.46 (1.16) | 2.64 (2.28-3.05) | <0.001 | <0.001 |
| cardiac failure chronic | Cardiac disorders | 160 | 70,646 | 28 | 134,451 | 10.88 (7.28-16.25) | 10.85 (7.26-16.21) | 213.35 | 1.28 (1.03) | 2.37 (2.09-2.69) | <0.001 | <0.001 |
| carotid arteriosclerosis | Nervous system disorders | 93 | 70,713 | 9 | 134,470 | 19.65 (9.91-38.95) | 19.63 (9.90-38.90) | 145.12 | 1.36 (1.02) | 2.46 (2.08-2.89) | <0.001 | <0.001 |
| carotid artery thrombosis | Nervous system disorders | 177 | 70,629 | 23 | 134,456 | 14.65 (9.49-22.62) | 14.62 (9.47-22.57) | 258.44 | 1.34 (1.09) | 2.47 (2.18-2.79) | <0.001 | <0.001 |
| catarrh | Respiratory, thoracic and mediastinal disorders | 121 | 70,685 | 1 | 134,478 | 230.20 (32.16-1647.58) | 229.81 (32.11-1644.71) | 226.08 | 1.49 (1.19) | 2.69 (2.32-3.11) | <0.001 | <0.001 |
| chromaturia | Renal and urinary disorders | 431 | 70,375 | 113 | 134,366 | 7.28 (5.92-8.96) | 7.24 (5.89-8.91) | 483.13 | 1.19 (1.04) | 2.27 (2.09-2.45) | <0.001 | <0.001 |
| colitis ulcerative | Gastrointestinal disorders | 303 | 70,503 | 76 | 134,403 | 7.60 (5.91-9.77) | 7.57 (5.89-9.74) | 347.22 | 1.20 (1.02) | 2.28 (2.07-2.50) | <0.001 | <0.001 |
| congenital cardiovascular anomaly | Congenital, familial and genetic disorders | 58 | 70,748 | 2 | 134,477 | 55.12 (13.46-225.70) | 55.08 (13.45-225.51) | 102.68 | 1.42 (0.99) | 2.48 (2.01-3.04) | <0.001 | <0.001 |
| congenital hiatus hernia | Congenital, familial and genetic disorders | 83 | 70,723 | 0 | 134,479 | 317.55 (19.70-5119.38) | Inf (19.67-5113.21) | 157.70 | 1.49 (1.12) | 2.64 (2.21-3.13) | <0.001 | <0.001 |
| coronary artery occlusion | Cardiac disorders | 543 | 70,263 | 147 | 134,332 | 7.06 (5.88-8.48) | 7.02 (5.85-8.42) | 598.72 | 1.18 (1.05) | 2.26 (2.11-2.42) | <0.001 | <0.001 |
| creatinine renal clearance decreased | Investigations | 256 | 70,550 | 63 | 134,416 | 7.74 (5.88-10.20) | 7.72 (5.86-10.17) | 296.08 | 1.21 (1.01) | 2.28 (2.05-2.52) | <0.001 | <0.001 |
| diabetes mellitus | Metabolism and nutrition disorders | 651 | 70,155 | 211 | 134,268 | 5.90 (5.05-6.90) | 5.86 (5.02-6.84) | 644.97 | 1.13 (1.00) | 2.17 (2.03-2.31) | <0.001 | <0.001 |
| diabetic neuropathy | Nervous system disorders | 112 | 70,694 | 13 | 134,466 | 16.39 (9.23-29.10) | 16.36 (9.21-29.06) | 168.11 | 1.35 (1.04) | 2.45 (2.10-2.84) | <0.001 | <0.001 |
| drug dose omission | Injury, poisoning and procedural complications | 420 | 70,386 | 105 | 134,374 | 7.64 (6.16-9.46) | 7.60 (6.14-9.41) | 482.44 | 1.21 (1.05) | 2.29 (2.11-2.48) | <0.001 | <0.001 |
| dyslipidaemia | Metabolism and nutrition disorders | 142 | 70,664 | 9 | 134,470 | 30.02 (15.31-58.90) | 29.97 (15.28-58.78) | 237.15 | 1.42 (1.14) | 2.59 (2.25-2.96) | <0.001 | <0.001 |
| eczema | Skin and subcutaneous tissue disorders | 380 | 70,426 | 116 | 134,363 | 6.25 (5.08-7.70) | 6.22 (5.05-7.66) | 390.42 | 1.14 (0.98) | 2.19 (2.02-2.38) | <0.001 | <0.001 |
| erectile dysfunction | Reproductive system and breast disorders | 322 | 70,484 | 48 | 134,431 | 12.79 (9.45-17.33) | 12.74 (9.41-17.25) | 452.77 | 1.32 (1.14) | 2.47 (2.25-2.70) | <0.001 | <0.001 |
| exercise tolerance decreased | General disorders and administration site conditions | 202 | 70,604 | 15 | 134,464 | 25.65 (15.18-43.34) | 25.58 (15.14-43.21) | 330.10 | 1.41 (1.18) | 2.60 (2.32-2.91) | <0.001 | <0.001 |
| feeling hot | General disorders and administration site conditions | 833 | 69,973 | 120 | 134,359 | 13.33 (11.01-16.14) | 13.18 (10.89-15.96) | 1186.56 | 1.34 (1.22) | 2.51 (2.37-2.66) | <0.001 | <0.001 |
| female genital tract fistula | Reproductive system and breast disorders | 130 | 70,676 | 14 | 134,465 | 17.67 (10.18-30.66) | 17.64 (10.16-30.61) | 198.48 | 1.36 (1.07) | 2.48 (2.15-2.86) | <0.001 | <0.001 |
| flushing | Vascular disorders | 2,531 | 68,275 | 188 | 134,291 | 26.48 (22.83-30.72) | 25.57 (22.05-29.64) | 4186.95 | 1.43 (1.37) | 2.69 (2.61-2.78) | <0.001 | <0.001 |
| fractional exhaled nitric oxide increased | Investigations | 94 | 70,712 | 0 | 134,479 | 359.44 (22.32-5789.11) | Inf (22.29-5781.23) | 178.61 | 1.49 (1.15) | 2.67 (2.25-3.14) | <0.001 | <0.001 |
| frequent bowel movements | Gastrointestinal disorders | 303 | 70,503 | 56 | 134,423 | 10.32 (7.76-13.72) | 10.28 (7.73-13.66) | 396.47 | 1.28 (1.09) | 2.40 (2.18-2.63) | <0.001 | <0.001 |
| gait spastic | Nervous system disorders | 59 | 70,747 | 0 | 134,479 | 226.20 (13.98-3658.88) | Inf (13.97-3655.71) | 112.09 | 1.47 (1.03) | 2.56 (2.08-3.12) | <0.001 | <0.001 |
| generalised oedema | General disorders and administration site conditions | 193 | 70,613 | 43 | 134,436 | 8.55 (6.14-11.89) | 8.52 (6.13-11.86) | 233.83 | 1.23 (1.00) | 2.30 (2.05-2.58) | <0.001 | <0.001 |
| gingival pain | Gastrointestinal disorders | 139 | 70,667 | 10 | 134,469 | 26.45 (13.92-50.25) | 26.40 (13.90-50.15) | 228.14 | 1.41 (1.13) | 2.57 (2.24-2.94) | <0.001 | <0.001 |
| glomerular filtration rate decreased | Investigations | 150 | 70,656 | 32 | 134,447 | 8.92 (6.09-13.07) | 8.90 (6.08-13.04) | 185.18 | 1.23 (0.97) | 2.30 (2.01-2.62) | <0.001 | <0.001 |
| glucose tolerance impaired | Metabolism and nutrition disorders | 80 | 70,726 | 7 | 134,472 | 21.73 (10.03-47.05) | 21.71 (10.03-47.00) | 127.19 | 1.37 (1.00) | 2.45 (2.05-2.92) | <0.001 | <0.001 |
| glycosylated haemoglobin increased | Investigations | 203 | 70,603 | 51 | 134,428 | 7.58 (5.57-10.30) | 7.56 (5.56-10.27) | 232.30 | 1.20 (0.97) | 2.25 (2.01-2.52) | <0.001 | <0.001 |
| gynaecomastia | Reproductive system and breast disorders | 120 | 70,686 | 11 | 134,468 | 20.75 (11.19-38.48) | 20.72 (11.17-38.41) | 189.23 | 1.38 (1.08) | 2.51 (2.16-2.90) | <0.001 | <0.001 |
| hepatic enzyme increased | Investigations | 537 | 70,269 | 174 | 134,305 | 5.90 (4.97-7.00) | 5.86 (4.94-6.95) | 531.73 | 1.13 (0.99) | 2.17 (2.02-2.33) | <0.001 | <0.001 |
| hepatitis cholestatic | Hepatobiliary disorders | 220 | 70,586 | 48 | 134,431 | 8.73 (6.39-11.93) | 8.70 (6.37-11.89) | 269.07 | 1.24 (1.02) | 2.32 (2.08-2.58) | <0.001 | <0.001 |
| hepatocellular injury | Hepatobiliary disorders | 391 | 70,415 | 119 | 134,360 | 6.27 (5.11-7.70) | 6.24 (5.08-7.66) | 402.49 | 1.14 (0.99) | 2.19 (2.02-2.38) | <0.001 | <0.001 |
| high density lipoprotein decreased | Investigations | 222 | 70,584 | 3 | 134,476 | 140.98 (45.12-440.50) | 140.55 (44.99-439.10) | 410.56 | 1.50 (1.27) | 2.76 (2.47-3.07) | <0.001 | <0.001 |
| hypercholesterolaemia | Metabolism and nutrition disorders | 225 | 70,581 | 50 | 134,429 | 8.57 (6.31-11.65) | 8.55 (6.29-11.61) | 272.97 | 1.23 (1.02) | 2.31 (2.07-2.57) | <0.001 | <0.001 |
| hypertensive emergency | Vascular disorders | 76 | 70,730 | 6 | 134,473 | 24.08 (10.49-55.29) | 24.06 (10.48-55.23) | 122.94 | 1.38 (1.00) | 2.46 (2.04-2.94) | <0.001 | <0.001 |
| hypertransaminasaemia | Hepatobiliary disorders | 110 | 70,696 | 14 | 134,465 | 14.94 (8.57-26.06) | 14.92 (8.56-26.02) | 161.42 | 1.33 (1.02) | 2.42 (2.08-2.81) | <0.001 | <0.001 |
| immune-mediated myositis | Musculoskeletal and connective tissue disorders | 128 | 70,678 | 1 | 134,478 | 243.54 (34.04-1742.30) | 243.11 (33.98-1739.09) | 239.39 | 1.49 (1.20) | 2.70 (2.34-3.11) | <0.001 | <0.001 |
| impaired quality of life | Social circumstances | 171 | 70,635 | 30 | 134,449 | 10.85 (7.36-15.99) | 10.83 (7.35-15.96) | 227.84 | 1.28 (1.04) | 2.38 (2.10-2.69) | <0.001 | <0.001 |
| impaired work ability | Social circumstances | 224 | 70,582 | 59 | 134,420 | 7.23 (5.43-9.63) | 7.21 (5.41-9.60) | 250.16 | 1.18 (0.97) | 2.24 (2.00-2.49) | <0.001 | <0.001 |
| interstitial lung disease | Respiratory, thoracic and mediastinal disorders | 594 | 70,212 | 177 | 134,302 | 6.42 (5.43-7.59) | 6.37 (5.39-7.54) | 620.16 | 1.15 (1.02) | 2.21 (2.07-2.37) | <0.001 | <0.001 |
| liver function test abnormal | Investigations | 594 | 70,212 | 89 | 134,390 | 12.77 (10.22-15.97) | 12.68 (10.15-15.84) | 835.23 | 1.33 (1.20) | 2.49 (2.33-2.67) | <0.001 | <0.001 |
| low density lipoprotein increased | Investigations | 251 | 70,555 | 33 | 134,446 | 14.49 (10.08-20.84) | 14.45 (10.05-20.76) | 365.52 | 1.34 (1.14) | 2.49 (2.25-2.76) | <0.001 | <0.001 |
| malignant melanoma in situ | Neoplasms benign, malignant and unspecified (incl cysts and polyps) | 82 | 70,724 | 0 | 134,479 | 313.74 (19.46-5058.50) | Inf (19.44-5052.48) | 155.80 | 1.49 (1.12) | 2.64 (2.21-3.13) | <0.001 | <0.001 |
| memory impairment | Nervous system disorders | 768 | 70,038 | 213 | 134,266 | 6.91 (5.94-8.05) | 6.85 (5.89-7.97) | 836.77 | 1.18 (1.06) | 2.25 (2.12-2.39) | <0.001 | <0.001 |
| micturition urgency | Renal and urinary disorders | 190 | 70,616 | 12 | 134,467 | 30.15 (16.82-54.04) | 30.07 (16.78-53.89) | 317.54 | 1.43 (1.19) | 2.62 (2.33-2.95) | <0.001 | <0.001 |
| mixed liver injury | Hepatobiliary disorders | 109 | 70,697 | 18 | 134,461 | 11.52 (6.99-18.96) | 11.50 (6.99-18.94) | 148.22 | 1.28 (0.97) | 2.35 (2.01-2.74) | <0.001 | <0.001 |
| mobility decreased | Musculoskeletal and connective tissue disorders | 597 | 70,209 | 126 | 134,353 | 9.07 (7.48-10.99) | 9.00 (7.43-10.90) | 742.35 | 1.25 (1.12) | 2.37 (2.21-2.53) | <0.001 | <0.001 |
| multiple drug therapy | Surgical and medical procedures | 84 | 70,722 | 5 | 134,474 | 31.94 (12.96-78.75) | 31.91 (12.94-78.65) | 141.35 | 1.41 (1.05) | 2.52 (2.11-2.98) | <0.001 | <0.001 |
| muscle atrophy | Musculoskeletal and connective tissue disorders | 282 | 70,524 | 27 | 134,452 | 19.91 (13.41-29.56) | 19.84 (13.37-29.44) | 441.42 | 1.39 (1.20) | 2.58 (2.34-2.84) | <0.001 | <0.001 |
| muscle disorder | Musculoskeletal and connective tissue disorders | 195 | 70,611 | 16 | 134,463 | 23.21 (13.94-38.64) | 23.15 (13.91-38.53) | 313.66 | 1.40 (1.17) | 2.58 (2.30-2.89) | <0.001 | <0.001 |
| muscle injury | Injury, poisoning and procedural complications | 128 | 70,678 | 18 | 134,461 | 13.53 (8.26-22.16) | 13.51 (8.25-22.12) | 182.87 | 1.32 (1.03) | 2.42 (2.09-2.78) | <0.001 | <0.001 |
| muscle rupture | Injury, poisoning and procedural complications | 126 | 70,680 | 18 | 134,461 | 13.32 (8.13-21.82) | 13.29 (8.11-21.78) | 179.20 | 1.32 (1.03) | 2.41 (2.08-2.77) | <0.001 | <0.001 |
| muscle spasms | Musculoskeletal and connective tissue disorders | 1,833 | 68,973 | 344 | 134,135 | 10.36 (9.23-11.63) | 10.12 (9.02-11.35) | 2406.09 | 1.29 (1.21) | 2.43 (2.34-2.53) | <0.001 | <0.001 |
| muscle spasticity | Nervous system disorders | 258 | 70,548 | 67 | 134,412 | 7.34 (5.61-9.60) | 7.31 (5.59-9.57) | 290.35 | 1.19 (0.99) | 2.25 (2.03-2.49) | <0.001 | <0.001 |
| muscular weakness | Musculoskeletal and connective tissue disorders | 2,079 | 68,727 | 394 | 134,085 | 10.29 (9.24-11.47) | 10.02 (9.00-11.16) | 2722.87 | 1.28 (1.21) | 2.43 (2.34-2.52) | <0.001 | <0.001 |
| musculoskeletal pain | Musculoskeletal and connective tissue disorders | 570 | 70,236 | 141 | 134,338 | 7.73 (6.43-9.30) | 7.68 (6.39-9.23) | 658.82 | 1.21 (1.08) | 2.30 (2.15-2.46) | <0.001 | <0.001 |
| myalgia | Musculoskeletal and connective tissue disorders | 4,219 | 66,587 | 316 | 134,163 | 26.90 (23.99-30.17) | 25.36 (22.63-28.42) | 7033.64 | 1.43 (1.38) | 2.69 (2.62-2.76) | <0.001 | <0.001 |
| myoglobin blood increased | Investigations | 181 | 70,625 | 11 | 134,468 | 31.33 (17.04-57.59) | 31.25 (17.00-57.43) | 303.95 | 1.43 (1.18) | 2.62 (2.32-2.95) | <0.001 | <0.001 |
| myopathy | Musculoskeletal and connective tissue disorders | 630 | 70,176 | 31 | 134,448 | 38.94 (27.15-55.85) | 38.60 (26.92-55.35) | 1085.59 | 1.46 (1.33) | 2.73 (2.55-2.91) | <0.001 | <0.001 |
| myositis | Musculoskeletal and connective tissue disorders | 432 | 70,374 | 11 | 134,468 | 75.04 (41.24-136.53) | 74.59 (41.00-135.69) | 780.48 | 1.49 (1.33) | 2.77 (2.56-3.00) | <0.001 | <0.001 |
| nasal oedema | Respiratory, thoracic and mediastinal disorders | 126 | 70,680 | 5 | 134,474 | 47.94 (19.61-117.20) | 47.86 (19.58-116.98) | 220.80 | 1.45 (1.15) | 2.63 (2.27-3.02) | <0.001 | <0.001 |
| necrotising myositis | Musculoskeletal and connective tissue disorders | 77 | 70,729 | 0 | 134,479 | 294.70 (18.27-4754.16) | Inf (18.25-4748.83) | 146.30 | 1.48 (1.10) | 2.62 (2.18-3.13) | <0.001 | <0.001 |
| needle fatigue | Injury, poisoning and procedural complications | 56 | 70,750 | 0 | 134,479 | 214.79 (13.27-3476.40) | Inf (13.26-3473.53) | 106.39 | 1.46 (1.02) | 2.54 (2.05-3.12) | <0.001 | <0.001 |
| neurologic neglect syndrome | Nervous system disorders | 173 | 70,633 | 22 | 134,457 | 14.97 (9.60-23.33) | 14.94 (9.58-23.27) | 254.01 | 1.34 (1.09) | 2.47 (2.18-2.79) | <0.001 | <0.001 |
| nocturia | Renal and urinary disorders | 188 | 70,618 | 36 | 134,443 | 9.94 (6.96-14.20) | 9.92 (6.94-14.17) | 242.56 | 1.27 (1.03) | 2.36 (2.09-2.65) | <0.001 | <0.001 |
| obstructive sleep apnoea syndrome | Respiratory, thoracic and mediastinal disorders | 92 | 70,714 | 2 | 134,477 | 87.48 (21.55-355.08) | 87.37 (21.53-354.60) | 167.20 | 1.46 (1.11) | 2.61 (2.21-3.08) | <0.001 | <0.001 |
| ocular discomfort | Eye disorders | 177 | 70,629 | 20 | 134,459 | 16.85 (10.61-26.75) | 16.81 (10.59-26.69) | 267.43 | 1.36 (1.12) | 2.51 (2.21-2.83) | <0.001 | <0.001 |
| oral candidiasis | Infections and infestations | 154 | 70,652 | 23 | 134,456 | 12.74 (8.22-19.75) | 12.72 (8.21-19.71) | 216.22 | 1.31 (1.05) | 2.42 (2.12-2.75) | <0.001 | <0.001 |
| orthostatic hypotension | Vascular disorders | 430 | 70,376 | 103 | 134,376 | 7.97 (6.43-9.89) | 7.93 (6.40-9.83) | 504.46 | 1.22 (1.06) | 2.31 (2.13-2.49) | <0.001 | <0.001 |
| pain in extremity | Musculoskeletal and connective tissue disorders | 2,446 | 68,360 | 746 | 133,733 | 6.41 (5.91-6.97) | 6.23 (5.74-6.76) | 2547.98 | 1.15 (1.09) | 2.22 (2.14-2.29) | <0.001 | <0.001 |
| pancreatitis acute | Gastrointestinal disorders | 399 | 70,407 | 123 | 134,356 | 6.19 (5.06-7.58) | 6.16 (5.03-7.54) | 407.50 | 1.14 (0.98) | 2.19 (2.02-2.37) | <0.001 | <0.001 |
| pancreatitis necrotising | Gastrointestinal disorders | 92 | 70,714 | 11 | 134,468 | 15.90 (8.51-29.72) | 15.88 (8.50-29.68) | 137.11 | 1.33 (0.99) | 2.41 (2.04-2.84) | <0.001 | <0.001 |
| paraesthesia | Nervous system disorders | 1,290 | 69,516 | 413 | 134,066 | 6.02 (5.39-6.73) | 5.93 (5.31-6.63) | 1293.66 | 1.13 (1.05) | 2.19 (2.09-2.29) | <0.001 | <0.001 |
| paraesthesia oral | Gastrointestinal disorders | 183 | 70,623 | 34 | 134,445 | 10.25 (7.10-14.78) | 10.22 (7.09-14.74) | 238.82 | 1.27 (1.03) | 2.36 (2.09-2.66) | <0.001 | <0.001 |
| parosmia | Nervous system disorders | 128 | 70,678 | 12 | 134,467 | 20.29 (11.23-36.68) | 20.26 (11.21-36.61) | 201.00 | 1.38 (1.09) | 2.51 (2.17-2.89) | <0.001 | <0.001 |
| personality disorder | Psychiatric disorders | 192 | 70,614 | 17 | 134,462 | 21.51 (13.10-35.32) | 21.45 (13.06-35.22) | 304.80 | 1.39 (1.16) | 2.57 (2.28-2.88) | <0.001 | <0.001 |
| plantar fasciitis | Musculoskeletal and connective tissue disorders | 116 | 70,690 | 5 | 134,474 | 44.13 (18.03-108.04) | 44.06 (18.00-107.86) | 201.85 | 1.44 (1.13) | 2.61 (2.24-3.02) | <0.001 | <0.001 |
| polymyositis | Musculoskeletal and connective tissue disorders | 104 | 70,702 | 2 | 134,477 | 98.91 (24.41-400.77) | 98.76 (24.37-400.16) | 189.99 | 1.47 (1.14) | 2.64 (2.25-3.08) | <0.001 | <0.001 |
| procedural pain | Injury, poisoning and procedural complications | 155 | 70,651 | 24 | 134,455 | 12.29 (7.99-18.89) | 12.27 (7.98-18.85) | 215.23 | 1.31 (1.05) | 2.41 (2.11-2.74) | <0.001 | <0.001 |
| proctitis | Gastrointestinal disorders | 132 | 70,674 | 13 | 134,466 | 19.32 (10.93-34.15) | 19.28 (10.91-34.09) | 205.32 | 1.37 (1.09) | 2.50 (2.17-2.87) | <0.001 | <0.001 |
| product substitution issue | Product issues | 167 | 70,639 | 32 | 134,447 | 9.93 (6.80-14.50) | 9.91 (6.79-14.47) | 215.38 | 1.26 (1.01) | 2.35 (2.07-2.65) | <0.001 | <0.001 |
| pruritus | Skin and subcutaneous tissue disorders | 2,351 | 68,455 | 909 | 133,570 | 5.05 (4.67-5.45) | 4.91 (4.55-5.30) | 2075.45 | 1.06 (1.00) | 2.09 (2.02-2.16) | <0.001 | <0.001 |
| pruritus genital | Reproductive system and breast disorders | 55 | 70,751 | 1 | 134,478 | 104.54 (14.47-755.46) | 104.46 (14.46-754.85) | 100.67 | 1.44 (0.99) | 2.50 (2.02-3.07) | <0.001 | <0.001 |
| radiculopathy | Nervous system disorders | 162 | 70,644 | 10 | 134,469 | 30.84 (16.28-58.41) | 30.77 (16.25-58.27) | 271.49 | 1.43 (1.17) | 2.61 (2.30-2.96) | <0.001 | <0.001 |
| red cell distribution width increased | Investigations | 71 | 70,735 | 5 | 134,474 | 27.00 (10.90-66.86) | 26.97 (10.89-66.79) | 116.85 | 1.38 (0.99) | 2.46 (2.03-2.96) | <0.001 | <0.001 |
| relapsing-remitting multiple sclerosis | Nervous system disorders | 56 | 70,750 | 1 | 134,478 | 106.44 (14.73-768.96) | 106.36 (14.72-768.33) | 102.57 | 1.44 (1.00) | 2.51 (2.02-3.08) | <0.001 | <0.001 |
| respiratory tract infection | Infections and infestations | 329 | 70,477 | 66 | 134,413 | 9.51 (7.30-12.39) | 9.47 (7.27-12.33) | 417.11 | 1.26 (1.08) | 2.37 (2.17-2.59) | <0.001 | <0.001 |
| rhabdomyolysis | Musculoskeletal and connective tissue disorders | 3,587 | 67,219 | 149 | 134,330 | 48.11 (40.83-56.69) | 45.72 (38.82-53.85) | 6373.96 | 1.48 (1.42) | 2.78 (2.70-2.86) | <0.001 | <0.001 |
| secretion discharge | General disorders and administration site conditions | 159 | 70,647 | 34 | 134,445 | 8.90 (6.14-12.89) | 8.88 (6.13-12.86) | 196.10 | 1.24 (0.98) | 2.30 (2.02-2.61) | <0.001 | <0.001 |
| sedation | Nervous system disorders | 274 | 70,532 | 49 | 134,430 | 10.66 (7.86-14.45) | 10.62 (7.84-14.39) | 362.80 | 1.29 (1.09) | 2.40 (2.18-2.65) | <0.001 | <0.001 |
| sedation complication | Injury, poisoning and procedural complications | 208 | 70,598 | 2 | 134,477 | 198.10 (49.21-797.43) | 197.52 (49.07-795.05) | 387.73 | 1.50 (1.27) | 2.76 (2.46-3.08) | <0.001 | <0.001 |
| sensory loss | Nervous system disorders | 225 | 70,581 | 45 | 134,434 | 9.52 (6.91-13.12) | 9.50 (6.90-13.08) | 285.44 | 1.26 (1.04) | 2.35 (2.11-2.62) | <0.001 | <0.001 |
| sinus congestion | Respiratory, thoracic and mediastinal disorders | 143 | 70,663 | 31 | 134,448 | 8.78 (5.95-12.94) | 8.76 (5.94-12.92) | 175.31 | 1.23 (0.96) | 2.29 (2.00-2.61) | <0.001 | <0.001 |
| skin burning sensation | Skin and subcutaneous tissue disorders | 369 | 70,437 | 69 | 134,410 | 10.20 (7.89-13.20) | 10.16 (7.86-13.13) | 480.91 | 1.28 (1.11) | 2.40 (2.21-2.61) | <0.001 | <0.001 |
| steroid dependence | General disorders and administration site conditions | 129 | 70,677 | 9 | 134,470 | 27.27 (13.87-53.60) | 27.22 (13.85-53.50) | 212.65 | 1.41 (1.12) | 2.56 (2.22-2.95) | <0.001 | <0.001 |
| tendon rupture | Injury, poisoning and procedural complications | 254 | 70,552 | 16 | 134,463 | 30.26 (18.25-50.15) | 30.15 (18.19-49.97) | 424.78 | 1.43 (1.23) | 2.65 (2.39-2.93) | <0.001 | <0.001 |
| tendonitis | Musculoskeletal and connective tissue disorders | 158 | 70,648 | 30 | 134,449 | 10.02 (6.78-14.81) | 10.00 (6.77-14.78) | 204.48 | 1.26 (1.01) | 2.35 (2.06-2.66) | <0.001 | <0.001 |
| transaminases increased | Investigations | 285 | 70,521 | 67 | 134,412 | 8.11 (6.21-10.58) | 8.08 (6.19-10.54) | 337.06 | 1.22 (1.03) | 2.30 (2.09-2.53) | <0.001 | <0.001 |
| truncus arteriosus persistent | Congenital, familial and genetic disorders | 67 | 70,739 | 0 | 134,479 | 256.64 (15.89-4145.60) | Inf (15.87-4141.54) | 127.29 | 1.48 (1.07) | 2.59 (2.13-3.12) | <0.001 | <0.001 |
| type 2 diabetes mellitus | Metabolism and nutrition disorders | 638 | 70,168 | 110 | 134,369 | 11.11 (9.07-13.60) | 11.02 (9.00-13.48) | 857.53 | 1.30 (1.17) | 2.45 (2.29-2.61) | <0.001 | <0.001 |
| urinary tract disorder | Renal and urinary disorders | 195 | 70,611 | 29 | 134,450 | 12.80 (8.67-18.91) | 12.77 (8.65-18.86) | 274.19 | 1.32 (1.09) | 2.44 (2.17-2.74) | <0.001 | <0.001 |
| vaginal discharge | Reproductive system and breast disorders | 134 | 70,672 | 11 | 134,468 | 23.18 (12.53-42.86) | 23.14 (12.51-42.78) | 215.45 | 1.39 (1.11) | 2.54 (2.21-2.92) | <0.001 | <0.001 |
| vaginal flatulence | Reproductive system and breast disorders | 123 | 70,683 | 3 | 134,476 | 78.00 (24.81-245.21) | 77.87 (24.77-244.77) | 222.36 | 1.47 (1.17) | 2.66 (2.30-3.07) | <0.001 | <0.001 |

**Note:** PT:Preferred Term; SOC:System Organ Class; a:Co-medicated reports with the PT; b:Co-medicated reports without the PT; c:Comparator reports with the PT; d:Comparator reports without the PT; ROR:Reporting Odds Ratio; PRR:Proportional Reporting Ratio; IC:Information Component; IC025:Lower 95% credibility limit of IC; EBGM: Empirical Bayes Geometric Mean; EB05:Lower 95% credibility limit of EBGM; EB95: Upper 95% credibility limit of EBGM; BH-FDR: Benjamini-Hochberg false discovery rate.

**Table S1.** Disproportionality signals: Co-medicated vs Aspirin without statins.
