# Supplementary material for: Disproportionality analysis of adverse event reporting with aspirin, statins, and their co-exposure in FAERS, 2004–2025
Source: Front Med (Lausanne). 2026 Oct 1;13:1923188. doi: 10.3389/fmed.2026.1923188 (PMC13669111; doi:10.3389/fmed.2026.1923188)
Supplement: Supplementary file 2 [file Table2.docx]

| **PT** | **Primary SOC** | **a** | **b** | **c** | **d** | **ROR (95% CI)** | **PRR (95% CI)** | **Chi-square** | **IC (IC025)** | **EBGM (EB05–EB95)** | **Fisher P** | **BH-FDR** |
| --- | --- | --- | --- | --- | --- | --- | --- | --- | --- | --- | --- | --- |
| accidental overdose | Injury, poisoning and procedural complications | 238 | 70,568 | 145 | 202,420 | 4.71 (3.83–5.79) | 4.70 (3.82–5.77) | 262.45 | 1.25 (1.05) | 2.37 (2.13–2.64) | <0.001 | <0.001 |
| acute coronary syndrome | Cardiac disorders | 224 | 70,582 | 140 | 202,425 | 4.59 (3.71–5.67) | 4.58 (3.71–5.65) | 241.19 | 1.23 (1.03) | 2.35 (2.10–2.62) | <0.001 | <0.001 |
| acute myocardial infarction | Cardiac disorders | 725 | 70,081 | 424 | 202,141 | 4.93 (4.37–5.56) | 4.89 (4.34–5.51) | 831.84 | 1.28 (1.17) | 2.43 (2.28–2.58) | <0.001 | <0.001 |
| adenocarcinoma of colon | Neoplasms benign, malignant and unspecified (incl cysts and polyps) | 30 | 70,776 | 8 | 202,557 | 10.73 (4.92–23.41) | 10.73 (4.92–23.40) | 55.72 | 1.48 (0.90) | 2.71 (2.00–3.60) | <0.001 | <0.001 |
| adjustment disorder | Psychiatric disorders | 198 | 70,608 | 14 | 202,551 | 40.57 (23.59–69.77) | 40.46 (23.53–69.57) | 503.60 | 1.82 (1.59) | 3.52 (3.12–3.95) | <0.001 | <0.001 |
| affect lability | Psychiatric disorders | 200 | 70,606 | 48 | 202,517 | 11.95 (8.72–16.38) | 11.92 (8.70–16.33) | 387.61 | 1.62 (1.39) | 3.05 (2.71–3.42) | <0.001 | <0.001 |
| alcohol poisoning | Injury, poisoning and procedural complications | 74 | 70,732 | 15 | 202,550 | 14.13 (8.11–24.61) | 14.11 (8.10–24.58) | 152.01 | 1.62 (1.25) | 3.04 (2.51–3.66) | <0.001 | <0.001 |
| altered state of consciousness | Nervous system disorders | 247 | 70,559 | 143 | 202,422 | 4.96 (4.03–6.09) | 4.94 (4.02–6.07) | 285.13 | 1.28 (1.08) | 2.42 (2.18–2.68) | <0.001 | <0.001 |
| amaurosis fugax | Eye disorders | 160 | 70,646 | 16 | 202,549 | 28.67 (17.15–47.94) | 28.61 (17.11–47.82) | 387.79 | 1.78 (1.52) | 3.41 (2.99–3.87) | <0.001 | <0.001 |
| anaemia | Blood and lymphatic system disorders | 3,101 | 67,705 | 1,411 | 201,154 | 6.53 (6.13–6.96) | 6.29 (5.91–6.69) | 4384.28 | 1.41 (1.35) | 2.65 (2.57–2.73) | <0.001 | <0.001 |
| angina pectoris | Cardiac disorders | 872 | 69,934 | 577 | 201,988 | 4.36 (3.93–4.85) | 4.32 (3.89–4.80) | 891.84 | 1.21 (1.11) | 2.32 (2.19–2.45) | <0.001 | <0.001 |
| angina unstable | Cardiac disorders | 251 | 70,555 | 130 | 202,435 | 5.54 (4.48–6.85) | 5.52 (4.47–6.83) | 317.72 | 1.33 (1.14) | 2.51 (2.26–2.79) | <0.001 | <0.001 |
| angiodysplasia | Vascular disorders | 19 | 70,787 | 1 | 202,564 | 54.37 (7.28–406.16) | 54.36 (7.28–406.04) | 49.76 | 1.63 (0.88) | 2.94 (2.02–4.16) | <0.001 | <0.001 |
| anosognosia | Nervous system disorders | 19 | 70,787 | 1 | 202,564 | 54.37 (7.28–406.16) | 54.36 (7.28–406.04) | 49.76 | 1.63 (0.88) | 2.94 (2.02–4.16) | <0.001 | <0.001 |
| aortic valve stenosis | Cardiac disorders | 76 | 70,730 | 18 | 202,547 | 12.09 (7.23–20.21) | 12.08 (7.23–20.19) | 147.94 | 1.59 (1.22) | 2.96 (2.45–3.56) | <0.001 | <0.001 |
| arterial haemorrhage | Vascular disorders | 50 | 70,756 | 6 | 202,559 | 23.86 (10.23–55.64) | 23.84 (10.22–55.60) | 117.25 | 1.69 (1.23) | 3.16 (2.50–3.95) | <0.001 | <0.001 |
| arteriovenous malformation | Congenital, familial and genetic disorders | 28 | 70,778 | 5 | 202,560 | 16.03 (6.19–41.51) | 16.02 (6.19–41.49) | 59.75 | 1.56 (0.96) | 2.85 (2.09–3.82) | <0.001 | <0.001 |
| atheroembolism | Vascular disorders | 22 | 70,784 | 2 | 202,563 | 31.48 (7.40–133.87) | 31.47 (7.40–133.83) | 54.09 | 1.62 (0.93) | 2.93 (2.06–4.07) | <0.001 | <0.001 |
| atrioventricular block second degree | Cardiac disorders | 54 | 70,752 | 17 | 202,548 | 9.09 (5.27–15.69) | 9.09 (5.27–15.67) | 93.08 | 1.48 (1.06) | 2.75 (2.20–3.41) | <0.001 | <0.001 |
| bezoar | Gastrointestinal disorders | 42 | 70,764 | 5 | 202,560 | 24.04 (9.51–60.78) | 24.03 (9.51–60.74) | 98.64 | 1.68 (1.18) | 3.11 (2.41–3.97) | <0.001 | <0.001 |
| bleeding time prolonged | Investigations | 45 | 70,761 | 10 | 202,555 | 12.88 (6.49–25.56) | 12.87 (6.49–25.54) | 89.62 | 1.57 (1.09) | 2.90 (2.27–3.67) | <0.001 | <0.001 |
| blood calcium decreased | Investigations | 265 | 70,541 | 140 | 202,425 | 5.43 (4.43–6.67) | 5.42 (4.41–6.64) | 330.25 | 1.32 (1.14) | 2.50 (2.25–2.76) | <0.001 | <0.001 |
| blood loss anaemia | Blood and lymphatic system disorders | 137 | 70,669 | 29 | 202,536 | 13.54 (9.07–20.21) | 13.52 (9.05–20.17) | 277.54 | 1.64 (1.37) | 3.09 (2.68–3.55) | <0.001 | <0.001 |
| brain contusion | Injury, poisoning and procedural complications | 28 | 70,778 | 4 | 202,561 | 20.03 (7.03–57.12) | 20.03 (7.02–57.09) | 63.27 | 1.60 (0.99) | 2.93 (2.14–3.92) | <0.001 | <0.001 |
| brain natriuretic peptide increased | Investigations | 140 | 70,666 | 54 | 202,511 | 7.43 (5.43–10.17) | 7.42 (5.42–10.15) | 216.50 | 1.45 (1.19) | 2.72 (2.36–3.11) | <0.001 | <0.001 |
| brain scan abnormal | Investigations | 35 | 70,771 | 5 | 202,560 | 20.04 (7.85–51.14) | 20.03 (7.85–51.11) | 79.09 | 1.63 (1.08) | 3.00 (2.27–3.91) | <0.001 | <0.001 |
| breath sounds abnormal | Investigations | 128 | 70,678 | 81 | 202,484 | 4.53 (3.43–5.98) | 4.52 (3.42–5.97) | 136.13 | 1.22 (0.95) | 2.32 (2.00–2.67) | <0.001 | <0.001 |
| bronchial obstruction | Respiratory, thoracic and mediastinal disorders | 28 | 70,778 | 5 | 202,560 | 16.03 (6.19–41.51) | 16.02 (6.19–41.49) | 59.75 | 1.56 (0.96) | 2.85 (2.09–3.82) | <0.001 | <0.001 |
| burns second degree | Injury, poisoning and procedural complications | 125 | 70,681 | 15 | 202,550 | 23.88 (13.98–40.80) | 23.84 (13.96–40.73) | 293.22 | 1.75 (1.46) | 3.32 (2.86–3.84) | <0.001 | <0.001 |
| cardiac murmur | Investigations | 201 | 70,605 | 137 | 202,428 | 4.21 (3.38–5.23) | 4.20 (3.38–5.21) | 198.67 | 1.18 (0.97) | 2.27 (2.02–2.54) | <0.001 | <0.001 |
| cardiac septal defect | Congenital, familial and genetic disorders | 53 | 70,753 | 4 | 202,561 | 37.93 (13.73–104.81) | 37.91 (13.72–104.73) | 133.67 | 1.75 (1.30) | 3.29 (2.62–4.09) | <0.001 | <0.001 |
| cardiac tamponade | Cardiac disorders | 56 | 70,750 | 13 | 202,552 | 12.33 (6.74–22.55) | 12.32 (6.74–22.53) | 109.81 | 1.57 (1.15) | 2.92 (2.34–3.61) | <0.001 | <0.001 |
| cardiorenal syndrome | Cardiac disorders | 24 | 70,782 | 3 | 202,562 | 22.89 (6.89–76.03) | 22.89 (6.89–76.00) | 55.82 | 1.60 (0.94) | 2.90 (2.08–3.97) | <0.001 | <0.001 |
| carotid artery stenosis | Nervous system disorders | 146 | 70,660 | 79 | 202,486 | 5.30 (4.03–6.96) | 5.29 (4.02–6.95) | 178.35 | 1.30 (1.05) | 2.46 (2.14–2.81) | <0.001 | <0.001 |
| carotid artery thrombosis | Nervous system disorders | 177 | 70,629 | 11 | 202,554 | 46.15 (25.10–84.85) | 46.03 (25.04–84.64) | 456.57 | 1.83 (1.58) | 3.54 (3.12–3.99) | <0.001 | <0.001 |
| catarrh | Respiratory, thoracic and mediastinal disorders | 121 | 70,685 | 20 | 202,545 | 17.34 (10.80–27.83) | 17.31 (10.78–27.78) | 263.86 | 1.69 (1.40) | 3.20 (2.75–3.70) | <0.001 | <0.001 |
| cerebellar haemorrhage | Nervous system disorders | 38 | 70,768 | 10 | 202,555 | 10.88 (5.42–21.83) | 10.87 (5.42–21.82) | 70.97 | 1.51 (0.99) | 2.78 (2.12–3.58) | <0.001 | <0.001 |
| cerebral artery thrombosis | Nervous system disorders | 33 | 70,773 | 0 | 202,565 | 191.77 (11.75–3129.89) | Inf (11.74–3128.33) | 94.42 | 1.79 (1.21) | 3.34 (2.50–4.38) | <0.001 | <0.001 |
| cerebral haematoma | Nervous system disorders | 165 | 70,641 | 80 | 202,485 | 5.91 (4.53–7.72) | 5.90 (4.52–7.71) | 219.48 | 1.36 (1.12) | 2.55 (2.24–2.89) | <0.001 | <0.001 |
| cerebral haemorrhage | Nervous system disorders | 931 | 69,875 | 356 | 202,209 | 7.57 (6.70–8.55) | 7.48 (6.62–8.45) | 1452.91 | 1.48 (1.38) | 2.78 (2.64–2.93) | <0.001 | <0.001 |
| chronic gastrointestinal bleeding | Gastrointestinal disorders | 18 | 70,788 | 0 | 202,565 | 105.88 (6.38–1757.00) | Inf (6.38–1756.50) | 51.50 | 1.68 (0.90) | 3.02 (2.05–4.32) | <0.001 | <0.001 |
| chronic sinusitis | Infections and infestations | 145 | 70,661 | 76 | 202,489 | 5.47 (4.14–7.22) | 5.46 (4.14–7.20) | 181.72 | 1.32 (1.06) | 2.48 (2.17–2.84) | <0.001 | <0.001 |
| coagulopathy | Blood and lymphatic system disorders | 251 | 70,555 | 161 | 202,404 | 4.47 (3.67–5.45) | 4.46 (3.66–5.44) | 263.69 | 1.22 (1.03) | 2.33 (2.09–2.58) | <0.001 | <0.001 |
| colitis ischaemic | Gastrointestinal disorders | 102 | 70,704 | 57 | 202,508 | 5.13 (3.71–7.09) | 5.12 (3.70–7.08) | 121.28 | 1.28 (0.98) | 2.41 (2.05–2.82) | <0.001 | <0.001 |
| colitis ulcerative | Gastrointestinal disorders | 303 | 70,503 | 167 | 202,398 | 5.21 (4.31–6.29) | 5.19 (4.30–6.27) | 364.88 | 1.30 (1.13) | 2.47 (2.24–2.71) | <0.001 | <0.001 |
| congenital cardiovascular anomaly | Congenital, familial and genetic disorders | 58 | 70,748 | 5 | 202,560 | 33.21 (13.32–82.81) | 33.19 (13.31–82.73) | 143.73 | 1.75 (1.32) | 3.28 (2.64–4.04) | <0.001 | <0.001 |
| congenital hiatus hernia | Congenital, familial and genetic disorders | 83 | 70,723 | 5 | 202,560 | 47.54 (19.28–117.24) | 47.49 (19.26–117.10) | 214.70 | 1.80 (1.44) | 3.44 (2.86–4.10) | <0.001 | <0.001 |
| coordination abnormal | Nervous system disorders | 257 | 70,549 | 152 | 202,413 | 4.85 (3.97–5.93) | 4.84 (3.96–5.91) | 291.15 | 1.27 (1.08) | 2.40 (2.17–2.65) | <0.001 | <0.001 |
| coronary artery disease | Cardiac disorders | 832 | 69,974 | 503 | 202,062 | 4.78 (4.27–5.34) | 4.73 (4.24–5.28) | 927.22 | 1.26 (1.16) | 2.40 (2.27–2.54) | <0.001 | <0.001 |
| coronary artery dissection | Cardiac disorders | 19 | 70,787 | 1 | 202,564 | 54.37 (7.28–406.16) | 54.36 (7.28–406.04) | 49.76 | 1.63 (0.88) | 2.94 (2.02–4.16) | <0.001 | <0.001 |
| coronary artery stenosis | Cardiac disorders | 253 | 70,553 | 104 | 202,461 | 6.98 (5.56–8.77) | 6.96 (5.54–8.74) | 376.62 | 1.44 (1.24) | 2.70 (2.43–2.99) | <0.001 | <0.001 |
| coronary artery thrombosis | Cardiac disorders | 47 | 70,759 | 15 | 202,550 | 8.97 (5.02–16.04) | 8.96 (5.01–16.03) | 80.47 | 1.47 (1.01) | 2.72 (2.14–3.42) | <0.001 | <0.001 |
| creatinine renal clearance decreased | Investigations | 256 | 70,550 | 82 | 202,483 | 8.96 (6.99–11.49) | 8.93 (6.97–11.45) | 437.98 | 1.53 (1.34) | 2.88 (2.60–3.19) | <0.001 | <0.001 |
| device dislocation | Product issues | 71 | 70,735 | 15 | 202,550 | 13.55 (7.77–23.66) | 13.54 (7.76–23.63) | 143.88 | 1.61 (1.23) | 3.01 (2.48–3.64) | <0.001 | <0.001 |
| dieulafoy's vascular malformation | Gastrointestinal disorders | 27 | 70,779 | 0 | 202,565 | 157.41 (9.60–2580.62) | Inf (9.60–2579.55) | 77.25 | 1.76 (1.12) | 3.24 (2.36–4.36) | <0.001 | <0.001 |
| diverticulum | Gastrointestinal disorders | 169 | 70,637 | 106 | 202,459 | 4.57 (3.58–5.83) | 4.56 (3.58–5.81) | 181.30 | 1.23 (1.00) | 2.34 (2.06–2.64) | <0.001 | <0.001 |
| diverticulum intestinal haemorrhagic | Gastrointestinal disorders | 184 | 70,622 | 7 | 202,558 | 75.39 (35.44–160.38) | 75.20 (35.35–159.95) | 494.05 | 1.87 (1.62) | 3.62 (3.20–4.08) | <0.001 | <0.001 |
| duodenal ulcer | Gastrointestinal disorders | 308 | 70,498 | 61 | 202,504 | 14.50 (11.02–19.09) | 14.44 (10.98–19.01) | 638.03 | 1.67 (1.49) | 3.18 (2.89–3.49) | <0.001 | <0.001 |
| duodenal ulcer haemorrhage | Gastrointestinal disorders | 151 | 70,655 | 18 | 202,547 | 24.05 (14.75–39.21) | 24.00 (14.72–39.12) | 354.70 | 1.75 (1.49) | 3.35 (2.92–3.82) | <0.001 | <0.001 |
| duodenitis | Gastrointestinal disorders | 143 | 70,663 | 32 | 202,533 | 12.81 (8.73–18.79) | 12.78 (8.71–18.75) | 284.22 | 1.63 (1.36) | 3.07 (2.67–3.51) | <0.001 | <0.001 |
| dyspnoea exertional | Respiratory, thoracic and mediastinal disorders | 755 | 70,051 | 556 | 202,009 | 3.92 (3.51–4.37) | 3.88 (3.48–4.33) | 689.23 | 1.15 (1.04) | 2.22 (2.09–2.35) | <0.001 | <0.001 |
| eating disorder | Psychiatric disorders | 245 | 70,561 | 171 | 202,394 | 4.11 (3.38–5.00) | 4.10 (3.37–4.98) | 236.31 | 1.17 (0.98) | 2.25 (2.02–2.50) | <0.001 | <0.001 |
| ecchymosis | Skin and subcutaneous tissue disorders | 150 | 70,656 | 67 | 202,498 | 6.42 (4.81–8.56) | 6.40 (4.80–8.54) | 211.40 | 1.39 (1.14) | 2.61 (2.28–2.98) | <0.001 | <0.001 |
| ecg signs of myocardial ischaemia | Investigations | 21 | 70,785 | 2 | 202,563 | 30.05 (7.05–128.15) | 30.04 (7.04–128.11) | 51.27 | 1.61 (0.90) | 2.90 (2.03–4.05) | <0.001 | <0.001 |
| electrocardiogram q wave abnormal | Investigations | 38 | 70,768 | 3 | 202,562 | 36.26 (11.19–117.46) | 36.24 (11.19–117.39) | 95.29 | 1.71 (1.18) | 3.18 (2.43–4.10) | <0.001 | <0.001 |
| electrocardiogram t wave inversion | Investigations | 72 | 70,734 | 19 | 202,546 | 10.85 (6.54–17.99) | 10.84 (6.54–17.97) | 134.34 | 1.55 (1.18) | 2.90 (2.39–3.49) | <0.001 | <0.001 |
| embolic stroke | Nervous system disorders | 56 | 70,750 | 13 | 202,552 | 12.33 (6.74–22.55) | 12.32 (6.74–22.53) | 109.81 | 1.57 (1.15) | 2.92 (2.34–3.61) | <0.001 | <0.001 |
| encephalitis toxic | Nervous system disorders | 19 | 70,787 | 1 | 202,564 | 54.37 (7.28–406.16) | 54.36 (7.28–406.04) | 49.76 | 1.63 (0.88) | 2.94 (2.02–4.16) | <0.001 | <0.001 |
| eosinophilic granulomatosis with polyangiitis | Immune system disorders | 50 | 70,756 | 8 | 202,557 | 17.89 (8.48–37.74) | 17.88 (8.48–37.71) | 109.93 | 1.65 (1.19) | 3.06 (2.42–3.83) | <0.001 | <0.001 |
| epistaxis | Respiratory, thoracic and mediastinal disorders | 1,316 | 69,490 | 646 | 201,919 | 5.92 (5.39–6.51) | 5.83 (5.31–6.40) | 1745.54 | 1.37 (1.29) | 2.58 (2.47–2.70) | <0.001 | <0.001 |
| erosive duodenitis | Gastrointestinal disorders | 43 | 70,763 | 5 | 202,560 | 24.62 (9.75–62.15) | 24.60 (9.75–62.11) | 101.44 | 1.68 (1.19) | 3.13 (2.43–3.98) | <0.001 | <0.001 |
| extradural haematoma | Injury, poisoning and procedural complications | 66 | 70,740 | 11 | 202,554 | 17.18 (9.07–32.53) | 17.17 (9.07–32.50) | 143.57 | 1.66 (1.26) | 3.11 (2.53–3.77) | <0.001 | <0.001 |
| facet joint syndrome | Musculoskeletal and connective tissue disorders | 21 | 70,785 | 2 | 202,563 | 30.05 (7.05–128.15) | 30.04 (7.04–128.11) | 51.27 | 1.61 (0.90) | 2.90 (2.03–4.05) | <0.001 | <0.001 |
| faeces discoloured | Gastrointestinal disorders | 445 | 70,361 | 281 | 202,284 | 4.55 (3.92–5.29) | 4.53 (3.90–5.26) | 475.13 | 1.24 (1.09) | 2.35 (2.18–2.54) | <0.001 | <0.001 |
| febrile bone marrow aplasia | Blood and lymphatic system disorders | 22 | 70,784 | 2 | 202,563 | 31.48 (7.40–133.87) | 31.47 (7.40–133.83) | 54.09 | 1.62 (0.93) | 2.93 (2.06–4.07) | <0.001 | <0.001 |
| female genital tract fistula | Reproductive system and breast disorders | 130 | 70,676 | 32 | 202,533 | 11.64 (7.91–17.14) | 11.62 (7.89–17.11) | 249.45 | 1.60 (1.32) | 3.01 (2.60–3.46) | <0.001 | <0.001 |
| femoral hernia incarcerated | Gastrointestinal disorders | 33 | 70,773 | 3 | 202,562 | 31.48 (9.66–102.66) | 31.47 (9.65–102.61) | 81.14 | 1.68 (1.11) | 3.10 (2.33–4.07) | <0.001 | <0.001 |
| fractional exhaled nitric oxide increased | Investigations | 94 | 70,712 | 0 | 202,565 | 541.42 (33.62–8720.07) | Inf (33.57–8708.23) | 269.01 | 1.89 (1.55) | 3.65 (3.08–4.31) | <0.001 | <0.001 |
| frequent bowel movements | Gastrointestinal disorders | 303 | 70,503 | 189 | 202,376 | 4.60 (3.84–5.52) | 4.59 (3.83–5.50) | 327.02 | 1.24 (1.07) | 2.36 (2.14–2.59) | <0.001 | <0.001 |
| gait spastic | Nervous system disorders | 59 | 70,747 | 23 | 202,542 | 7.34 (4.54–11.89) | 7.34 (4.53–11.88) | 90.63 | 1.41 (1.01) | 2.63 (2.12–3.23) | <0.001 | <0.001 |
| gangrene | Infections and infestations | 103 | 70,703 | 38 | 202,527 | 7.76 (5.35–11.26) | 7.75 (5.35–11.25) | 163.40 | 1.46 (1.15) | 2.73 (2.31–3.20) | <0.001 | <0.001 |
| gastric haemorrhage | Gastrointestinal disorders | 178 | 70,628 | 69 | 202,496 | 7.40 (5.60–9.77) | 7.38 (5.59–9.74) | 274.51 | 1.46 (1.22) | 2.73 (2.41–3.08) | <0.001 | <0.001 |
| gastric ulcer | Gastrointestinal disorders | 494 | 70,312 | 174 | 202,391 | 8.17 (6.87–9.72) | 8.12 (6.83–9.65) | 805.59 | 1.51 (1.36) | 2.83 (2.63–3.05) | <0.001 | <0.001 |
| gastric ulcer haemorrhage | Gastrointestinal disorders | 287 | 70,519 | 38 | 202,527 | 21.69 (15.46–30.43) | 21.61 (15.41–30.30) | 660.29 | 1.75 (1.56) | 3.36 (3.04–3.69) | <0.001 | <0.001 |
| gastric ulcer perforation | Gastrointestinal disorders | 28 | 70,778 | 4 | 202,561 | 20.03 (7.03–57.12) | 20.03 (7.02–57.09) | 63.27 | 1.60 (0.99) | 2.93 (2.14–3.92) | <0.001 | <0.001 |
| gastritis erosive | Gastrointestinal disorders | 215 | 70,591 | 28 | 202,537 | 22.03 (14.86–32.67) | 21.97 (14.82–32.57) | 496.23 | 1.75 (1.53) | 3.34 (2.99–3.73) | <0.001 | <0.001 |
| gastroduodenal ulcer | Gastrointestinal disorders | 23 | 70,783 | 3 | 202,562 | 21.94 (6.59–73.07) | 21.93 (6.59–73.05) | 53.03 | 1.58 (0.91) | 2.87 (2.04–3.95) | <0.001 | <0.001 |
| gastrointestinal angiodysplasia | Gastrointestinal disorders | 39 | 70,767 | 0 | 202,565 | 226.13 (13.90–3679.28) | Inf (13.89–3677.14) | 111.59 | 1.82 (1.28) | 3.40 (2.61–4.38) | <0.001 | <0.001 |
| gastrointestinal angiodysplasia haemorrhagic | Gastrointestinal disorders | 28 | 70,778 | 0 | 202,565 | 163.13 (9.96–2672.15) | Inf (9.96–2671.01) | 80.11 | 1.77 (1.14) | 3.26 (2.39–4.37) | <0.001 | <0.001 |
| gastrointestinal arteriovenous malformation | Congenital, familial and genetic disorders | 35 | 70,771 | 2 | 202,563 | 50.09 (12.05–208.26) | 50.06 (12.04–208.15) | 90.98 | 1.73 (1.17) | 3.21 (2.43–4.17) | <0.001 | <0.001 |
| gastrointestinal haemorrhage | Gastrointestinal disorders | 3,460 | 67,346 | 354 | 202,211 | 29.35 (26.30–32.75) | 27.96 (25.07–31.18) | 8467.11 | 1.81 (1.75) | 3.50 (3.40–3.60) | <0.001 | <0.001 |
| gastrointestinal polyp haemorrhage | Gastrointestinal disorders | 22 | 70,784 | 1 | 202,564 | 62.96 (8.49–467.10) | 62.94 (8.48–466.94) | 58.31 | 1.67 (0.97) | 3.03 (2.14–4.20) | <0.001 | <0.001 |
| gastrointestinal ulcer | Gastrointestinal disorders | 49 | 70,757 | 15 | 202,550 | 9.35 (5.24–16.68) | 9.35 (5.24–16.66) | 85.61 | 1.48 (1.04) | 2.75 (2.17–3.45) | <0.001 | <0.001 |
| gastrointestinal ulcer haemorrhage | Gastrointestinal disorders | 40 | 70,766 | 1 | 202,564 | 114.50 (15.74–832.91) | 114.43 (15.73–832.41) | 109.72 | 1.79 (1.26) | 3.34 (2.57–4.28) | <0.001 | <0.001 |
| gastrointestinal vascular malformation haemorrhagic | Gastrointestinal disorders | 54 | 70,752 | 0 | 202,565 | 312.07 (19.27–5053.22) | Inf (19.26–5049.21) | 154.52 | 1.85 (1.40) | 3.52 (2.81–4.36) | <0.001 | <0.001 |
| gingival bleeding | Gastrointestinal disorders | 193 | 70,613 | 131 | 202,434 | 4.22 (3.38–5.27) | 4.21 (3.38–5.26) | 191.57 | 1.19 (0.97) | 2.27 (2.02–2.55) | <0.001 | <0.001 |
| gingival pain | Gastrointestinal disorders | 139 | 70,667 | 62 | 202,503 | 6.42 (4.76–8.67) | 6.41 (4.76–8.65) | 196.07 | 1.39 (1.13) | 2.61 (2.27–2.99) | <0.001 | <0.001 |
| haemarthrosis | Musculoskeletal and connective tissue disorders | 103 | 70,703 | 34 | 202,531 | 8.68 (5.89–12.79) | 8.67 (5.88–12.77) | 173.45 | 1.50 (1.19) | 2.80 (2.38–3.29) | <0.001 | <0.001 |
| haematemesis | Gastrointestinal disorders | 864 | 69,942 | 156 | 202,409 | 16.03 (13.51–19.01) | 15.84 (13.36–18.79) | 1844.68 | 1.70 (1.59) | 3.25 (3.08–3.44) | <0.001 | <0.001 |
| haematochezia | Gastrointestinal disorders | 1,365 | 69,441 | 421 | 202,144 | 9.44 (8.46–10.53) | 9.28 (8.32–10.34) | 2391.34 | 1.56 (1.47) | 2.94 (2.81–3.08) | <0.001 | <0.001 |
| haematoma | Vascular disorders | 616 | 70,190 | 411 | 202,154 | 4.32 (3.81–4.89) | 4.29 (3.79–4.86) | 623.82 | 1.21 (1.09) | 2.31 (2.16–2.46) | <0.001 | <0.001 |
| haematoma muscle | Musculoskeletal and connective tissue disorders | 63 | 70,743 | 13 | 202,552 | 13.88 (7.64–25.21) | 13.86 (7.63–25.19) | 128.66 | 1.61 (1.21) | 3.00 (2.44–3.67) | <0.001 | <0.001 |
| haematuria | Renal and urinary disorders | 1,132 | 69,674 | 571 | 201,994 | 5.75 (5.20–6.36) | 5.67 (5.13–6.27) | 1469.62 | 1.36 (1.27) | 2.56 (2.44–2.69) | <0.001 | <0.001 |
| haemoglobin decreased | Investigations | 1,092 | 69,714 | 807 | 201,758 | 3.92 (3.57–4.29) | 3.87 (3.54–4.24) | 995.12 | 1.15 (1.06) | 2.22 (2.11–2.33) | <0.001 | <0.001 |
| haemoperitoneum | Gastrointestinal disorders | 22 | 70,784 | 3 | 202,562 | 20.99 (6.28–70.12) | 20.98 (6.28–70.09) | 50.24 | 1.57 (0.88) | 2.84 (2.00–3.94) | <0.001 | <0.001 |
| haemoptysis | Respiratory, thoracic and mediastinal disorders | 507 | 70,299 | 268 | 202,297 | 5.44 (4.69–6.31) | 5.41 (4.67–6.27) | 632.41 | 1.33 (1.19) | 2.51 (2.33–2.70) | <0.001 | <0.001 |
| haemorrhage | Vascular disorders | 879 | 69,927 | 625 | 201,940 | 4.06 (3.66–4.50) | 4.02 (3.63–4.46) | 834.51 | 1.17 (1.07) | 2.25 (2.13–2.38) | <0.001 | <0.001 |
| haemorrhage intracranial | Nervous system disorders | 287 | 70,519 | 154 | 202,411 | 5.35 (4.40–6.51) | 5.33 (4.38–6.48) | 353.27 | 1.32 (1.14) | 2.49 (2.25–2.74) | <0.001 | <0.001 |
| haemorrhage subcutaneous | Skin and subcutaneous tissue disorders | 74 | 70,732 | 38 | 202,527 | 5.58 (3.77–8.25) | 5.57 (3.77–8.24) | 94.21 | 1.31 (0.95) | 2.45 (2.02–2.95) | <0.001 | <0.001 |
| haemorrhage urinary tract | Renal and urinary disorders | 79 | 70,727 | 17 | 202,548 | 13.31 (7.88–22.48) | 13.29 (7.87–22.45) | 159.11 | 1.61 (1.25) | 3.02 (2.51–3.61) | <0.001 | <0.001 |
| haemorrhagic anaemia | Blood and lymphatic system disorders | 425 | 70,381 | 15 | 202,550 | 81.54 (48.72–136.47) | 81.06 (48.44–135.64) | 1147.46 | 1.89 (1.73) | 3.68 (3.40–3.99) | <0.001 | <0.001 |
| haemorrhagic arteriovenous malformation | Congenital, familial and genetic disorders | 96 | 70,710 | 0 | 202,565 | 552.89 (34.33–8903.53) | Inf (34.29–8891.18) | 274.74 | 1.89 (1.55) | 3.66 (3.09–4.31) | <0.001 | <0.001 |
| haemorrhagic cerebral infarction | Nervous system disorders | 25 | 70,781 | 1 | 202,564 | 71.55 (9.69–528.04) | 71.52 (9.69–527.84) | 66.87 | 1.70 (1.04) | 3.11 (2.24–4.23) | <0.001 | <0.001 |
| haemorrhagic erosive gastritis | Gastrointestinal disorders | 26 | 70,780 | 0 | 202,565 | 151.68 (9.24–2489.09) | Inf (9.24–2488.10) | 74.39 | 1.76 (1.10) | 3.22 (2.33–4.36) | <0.001 | <0.001 |
| haemorrhagic stroke | Nervous system disorders | 438 | 70,368 | 105 | 202,460 | 12.00 (9.70–14.85) | 11.93 (9.65–14.76) | 850.14 | 1.63 (1.48) | 3.09 (2.85–3.33) | <0.001 | <0.001 |
| haemorrhagic transformation stroke | Nervous system disorders | 79 | 70,727 | 8 | 202,557 | 28.28 (13.67–58.53) | 28.25 (13.65–58.46) | 191.01 | 1.75 (1.38) | 3.31 (2.74–3.96) | <0.001 | <0.001 |
| haemorrhoidal haemorrhage | Gastrointestinal disorders | 82 | 70,724 | 29 | 202,536 | 8.10 (5.30–12.37) | 8.09 (5.30–12.35) | 133.16 | 1.47 (1.12) | 2.73 (2.28–3.26) | <0.001 | <0.001 |
| heart disease congenital | Congenital, familial and genetic disorders | 65 | 70,741 | 9 | 202,556 | 20.68 (10.30–41.53) | 20.66 (10.29–41.48) | 147.95 | 1.69 (1.29) | 3.17 (2.58–3.86) | <0.001 | <0.001 |
| helicobacter gastritis | Infections and infestations | 57 | 70,749 | 18 | 202,547 | 9.07 (5.34–15.40) | 9.06 (5.33–15.39) | 98.11 | 1.48 (1.07) | 2.76 (2.21–3.40) | <0.001 | <0.001 |
| helicobacter test positive | Investigations | 39 | 70,767 | 4 | 202,561 | 27.91 (9.97–78.10) | 27.89 (9.97–78.05) | 94.08 | 1.69 (1.17) | 3.13 (2.40–4.02) | <0.001 | <0.001 |
| hemiparesis | Nervous system disorders | 354 | 70,452 | 174 | 202,391 | 5.84 (4.87–7.01) | 5.82 (4.86–6.98) | 466.62 | 1.36 (1.20) | 2.57 (2.35–2.80) | <0.001 | <0.001 |
| hemiplegia | Nervous system disorders | 331 | 70,475 | 121 | 202,444 | 7.86 (6.38–9.68) | 7.83 (6.36–9.64) | 528.42 | 1.49 (1.32) | 2.80 (2.55–3.06) | <0.001 | <0.001 |
| hepatojugular reflux | Cardiac disorders | 45 | 70,761 | 6 | 202,559 | 21.47 (9.16–50.33) | 21.46 (9.15–50.29) | 103.27 | 1.67 (1.18) | 3.10 (2.42–3.92) | <0.001 | <0.001 |
| hypochromasia | Blood and lymphatic system disorders | 17 | 70,789 | 0 | 202,565 | 100.15 (6.02–1665.52) | Inf (6.02–1665.07) | 48.64 | 1.67 (0.87) | 2.98 (2.00–4.31) | <0.001 | <0.001 |
| hypochromic anaemia | Blood and lymphatic system disorders | 37 | 70,769 | 4 | 202,561 | 26.48 (9.44–74.28) | 26.46 (9.43–74.24) | 88.45 | 1.68 (1.14) | 3.10 (2.36–4.01) | <0.001 | <0.001 |
| hypocoagulable state | Blood and lymphatic system disorders | 31 | 70,775 | 8 | 202,557 | 11.09 (5.10–24.13) | 11.09 (5.10–24.12) | 58.36 | 1.49 (0.92) | 2.73 (2.03–3.61) | <0.001 | <0.001 |
| hypopnoea | Respiratory, thoracic and mediastinal disorders | 124 | 70,682 | 20 | 202,545 | 17.77 (11.08–28.49) | 17.74 (11.06–28.44) | 272.14 | 1.70 (1.41) | 3.21 (2.77–3.71) | <0.001 | <0.001 |
| impaired quality of life | Social circumstances | 171 | 70,635 | 52 | 202,513 | 9.43 (6.91–12.86) | 9.41 (6.90–12.83) | 299.86 | 1.54 (1.30) | 2.90 (2.55–3.28) | <0.001 | <0.001 |
| in-stent coronary artery restenosis | General disorders and administration site conditions | 18 | 70,788 | 0 | 202,565 | 105.88 (6.38–1757.00) | Inf (6.38–1756.50) | 51.50 | 1.68 (0.90) | 3.02 (2.05–4.32) | <0.001 | <0.001 |
| incorrect drug administration duration | Injury, poisoning and procedural complications | 91 | 70,715 | 31 | 202,534 | 8.41 (5.59–12.64) | 8.40 (5.59–12.62) | 150.76 | 1.48 (1.16) | 2.77 (2.33–3.27) | <0.001 | <0.001 |
| infective exacerbation of chronic obstructive airways disease | Infections and infestations | 24 | 70,782 | 2 | 202,563 | 34.34 (8.12–145.31) | 34.33 (8.11–145.26) | 59.75 | 1.64 (0.98) | 2.99 (2.14–4.09) | <0.001 | <0.001 |
| inspiratory capacity abnormal | Investigations | 17 | 70,789 | 0 | 202,565 | 100.15 (6.02–1665.52) | Inf (6.02–1665.07) | 48.64 | 1.67 (0.87) | 2.98 (2.00–4.31) | <0.001 | <0.001 |
| internal haemorrhage | Vascular disorders | 324 | 70,482 | 24 | 202,541 | 38.79 (25.62–58.73) | 38.62 (25.51–58.46) | 819.92 | 1.83 (1.65) | 3.54 (3.23–3.88) | <0.001 | <0.001 |
| intracranial haematoma | Nervous system disorders | 25 | 70,781 | 0 | 202,565 | 145.95 (8.89–2397.56) | Inf (8.88–2396.64) | 71.53 | 1.75 (1.08) | 3.20 (2.31–4.36) | <0.001 | <0.001 |
| iron deficiency anaemia | Blood and lymphatic system disorders | 266 | 70,540 | 81 | 202,484 | 9.43 (7.35–12.09) | 9.39 (7.33–12.05) | 466.37 | 1.55 (1.36) | 2.92 (2.64–3.23) | <0.001 | <0.001 |
| ischaemic cardiomyopathy | Cardiac disorders | 107 | 70,699 | 28 | 202,537 | 10.95 (7.22–16.60) | 10.93 (7.21–16.57) | 200.36 | 1.57 (1.27) | 2.95 (2.52–3.45) | <0.001 | <0.001 |
| ischaemic stroke | Nervous system disorders | 432 | 70,374 | 257 | 202,308 | 4.83 (4.14–5.64) | 4.81 (4.12–5.61) | 487.36 | 1.27 (1.12) | 2.41 (2.22–2.60) | <0.001 | <0.001 |
| kounis syndrome | Cardiac disorders | 26 | 70,780 | 2 | 202,563 | 37.20 (8.83–156.76) | 37.19 (8.83–156.69) | 65.41 | 1.66 (1.02) | 3.04 (2.20–4.11) | <0.001 | <0.001 |
| labelled drug-drug interaction medication error | Injury, poisoning and procedural complications | 370 | 70,436 | 251 | 202,314 | 4.23 (3.61–4.97) | 4.22 (3.59–4.95) | 367.88 | 1.19 (1.04) | 2.28 (2.09–2.49) | <0.001 | <0.001 |
| labile blood pressure | Vascular disorders | 58 | 70,748 | 14 | 202,551 | 11.86 (6.62–21.26) | 11.85 (6.61–21.24) | 112.09 | 1.57 (1.15) | 2.91 (2.34–3.58) | <0.001 | <0.001 |
| large intestinal haemorrhage | Gastrointestinal disorders | 57 | 70,749 | 9 | 202,556 | 18.13 (8.98–36.63) | 18.12 (8.97–36.60) | 125.75 | 1.66 (1.23) | 3.10 (2.49–3.82) | <0.001 | <0.001 |
| large intestinal ulcer | Gastrointestinal disorders | 42 | 70,764 | 6 | 202,559 | 20.04 (8.52–47.14) | 20.03 (8.51–47.11) | 94.91 | 1.65 (1.15) | 3.06 (2.37–3.89) | <0.001 | <0.001 |
| left ventricular failure | Cardiac disorders | 92 | 70,714 | 40 | 202,525 | 6.59 (4.54–9.55) | 6.58 (4.54–9.54) | 131.98 | 1.39 (1.07) | 2.60 (2.18–3.07) | <0.001 | <0.001 |
| low birth weight baby | Pregnancy, puerperium and perinatal conditions | 97 | 70,709 | 30 | 202,535 | 9.26 (6.15–13.95) | 9.25 (6.14–13.93) | 168.68 | 1.52 (1.20) | 2.84 (2.40–3.34) | <0.001 | <0.001 |
| lower gastrointestinal haemorrhage | Gastrointestinal disorders | 388 | 70,418 | 40 | 202,525 | 27.90 (20.14–38.63) | 27.75 (20.04–38.42) | 936.52 | 1.79 (1.63) | 3.46 (3.18–3.76) | <0.001 | <0.001 |
| lung diffusion test decreased | Investigations | 24 | 70,782 | 0 | 202,565 | 140.23 (8.53–2306.03) | Inf (8.52–2305.18) | 68.67 | 1.74 (1.06) | 3.18 (2.27–4.36) | <0.001 | <0.001 |
| lymphatic fistula | Vascular disorders | 32 | 70,774 | 0 | 202,565 | 186.04 (11.39–3038.33) | Inf (11.39–3036.87) | 91.56 | 1.79 (1.20) | 3.32 (2.48–4.38) | <0.001 | <0.001 |
| malignant melanoma in situ | Neoplasms benign, malignant and unspecified (incl cysts and polyps) | 82 | 70,724 | 5 | 202,560 | 46.97 (19.04–115.87) | 46.92 (19.02–115.73) | 211.85 | 1.80 (1.44) | 3.43 (2.86–4.10) | <0.001 | <0.001 |
| malignant spinal cord compression | Nervous system disorders | 20 | 70,786 | 1 | 202,564 | 57.23 (7.68–426.47) | 57.22 (7.68–426.34) | 52.61 | 1.65 (0.91) | 2.97 (2.06–4.18) | <0.001 | <0.001 |
| mallory-weiss syndrome | Gastrointestinal disorders | 48 | 70,758 | 6 | 202,559 | 22.90 (9.80–53.51) | 22.89 (9.80–53.48) | 111.65 | 1.68 (1.21) | 3.14 (2.47–3.94) | <0.001 | <0.001 |
| melaena | Gastrointestinal disorders | 1,114 | 69,692 | 171 | 202,394 | 18.92 (16.10–22.23) | 18.64 (15.87–21.89) | 2486.03 | 1.74 (1.64) | 3.33 (3.17–3.50) | <0.001 | <0.001 |
| microcytic anaemia | Blood and lymphatic system disorders | 87 | 70,719 | 25 | 202,540 | 9.97 (6.39–15.55) | 9.96 (6.38–15.53) | 156.51 | 1.54 (1.20) | 2.87 (2.40–3.41) | <0.001 | <0.001 |
| micturition urgency | Renal and urinary disorders | 190 | 70,616 | 131 | 202,434 | 4.16 (3.33–5.20) | 4.15 (3.32–5.18) | 185.56 | 1.18 (0.96) | 2.25 (2.00–2.53) | <0.001 | <0.001 |
| mitral valve incompetence | Cardiac disorders | 302 | 70,504 | 215 | 202,350 | 4.03 (3.38–4.80) | 4.02 (3.37–4.79) | 285.29 | 1.16 (0.99) | 2.24 (2.03–2.46) | <0.001 | <0.001 |
| mitral valve thickening | Cardiac disorders | 31 | 70,775 | 2 | 202,563 | 44.36 (10.62–185.37) | 44.34 (10.61–185.28) | 79.61 | 1.70 (1.12) | 3.14 (2.34–4.16) | <0.001 | <0.001 |
| monoclonal b-cell lymphocytosis | Blood and lymphatic system disorders | 20 | 70,786 | 0 | 202,565 | 117.33 (7.10–1939.99) | Inf (7.09–1939.38) | 57.22 | 1.71 (0.96) | 3.08 (2.14–4.33) | <0.001 | <0.001 |
| monoclonal immunoglobulin present | Investigations | 26 | 70,780 | 3 | 202,562 | 24.80 (7.51–81.95) | 24.79 (7.50–81.91) | 61.42 | 1.62 (0.98) | 2.96 (2.14–4.00) | <0.001 | <0.001 |
| motor dysfunction | Nervous system disorders | 205 | 70,601 | 142 | 202,423 | 4.14 (3.34–5.13) | 4.13 (3.34–5.11) | 199.26 | 1.18 (0.97) | 2.25 (2.00–2.52) | <0.001 | <0.001 |
| muscle haemorrhage | Musculoskeletal and connective tissue disorders | 118 | 70,688 | 62 | 202,503 | 5.45 (4.01–7.42) | 5.44 (4.00–7.40) | 147.58 | 1.31 (1.03) | 2.47 (2.12–2.86) | <0.001 | <0.001 |
| muscle spasticity | Nervous system disorders | 258 | 70,548 | 96 | 202,469 | 7.71 (6.10–9.75) | 7.69 (6.08–9.72) | 407.63 | 1.48 (1.28) | 2.78 (2.51–3.07) | <0.001 | <0.001 |
| mycobacterium test positive | Investigations | 23 | 70,783 | 0 | 202,565 | 134.50 (8.17–2214.52) | Inf (8.17–2213.73) | 65.80 | 1.73 (1.04) | 3.16 (2.24–4.35) | <0.001 | <0.001 |
| myocardial ischaemia | Cardiac disorders | 264 | 70,542 | 163 | 202,402 | 4.65 (3.82–5.65) | 4.63 (3.81–5.63) | 287.60 | 1.24 (1.06) | 2.36 (2.14–2.61) | <0.001 | <0.001 |
| naevus flammeus | Congenital, familial and genetic disorders | 17 | 70,789 | 0 | 202,565 | 100.15 (6.02–1665.52) | Inf (6.02–1665.07) | 48.64 | 1.67 (0.87) | 2.98 (2.00–4.31) | <0.001 | <0.001 |
| nasal obstruction | Respiratory, thoracic and mediastinal disorders | 47 | 70,759 | 6 | 202,559 | 22.42 (9.59–52.45) | 22.41 (9.58–52.41) | 108.85 | 1.68 (1.20) | 3.12 (2.45–3.94) | <0.001 | <0.001 |
| nasal oedema | Respiratory, thoracic and mediastinal disorders | 126 | 70,680 | 27 | 202,538 | 13.37 (8.82–20.27) | 13.35 (8.81–20.23) | 254.19 | 1.63 (1.35) | 3.08 (2.65–3.55) | <0.001 | <0.001 |
| needle fatigue | Injury, poisoning and procedural complications | 56 | 70,750 | 2 | 202,563 | 80.17 (19.56–328.52) | 80.10 (19.55–328.24) | 150.88 | 1.81 (1.36) | 3.42 (2.74–4.23) | <0.001 | <0.001 |
| neurologic neglect syndrome | Nervous system disorders | 173 | 70,633 | 7 | 202,558 | 70.87 (33.29–150.90) | 70.70 (33.21–150.52) | 462.62 | 1.86 (1.61) | 3.60 (3.18–4.07) | <0.001 | <0.001 |
| nicotine dependence | Psychiatric disorders | 23 | 70,783 | 3 | 202,562 | 21.94 (6.59–73.07) | 21.93 (6.59–73.05) | 53.03 | 1.58 (0.91) | 2.87 (2.04–3.95) | <0.001 | <0.001 |
| obstructive sleep apnoea syndrome | Respiratory, thoracic and mediastinal disorders | 92 | 70,714 | 53 | 202,512 | 4.97 (3.55–6.97) | 4.97 (3.54–6.96) | 106.57 | 1.26 (0.94) | 2.38 (2.00–2.81) | <0.001 | <0.001 |
| occult blood | Investigations | 65 | 70,741 | 2 | 202,563 | 93.06 (22.79–380.08) | 92.98 (22.77–379.71) | 176.59 | 1.83 (1.41) | 3.47 (2.83–4.23) | <0.001 | <0.001 |
| occult blood positive | Investigations | 83 | 70,723 | 12 | 202,553 | 19.81 (10.81–36.29) | 19.79 (10.80–36.25) | 187.08 | 1.70 (1.34) | 3.20 (2.67–3.82) | <0.001 | <0.001 |
| ocular discomfort | Eye disorders | 177 | 70,629 | 48 | 202,517 | 10.57 (7.68–14.55) | 10.55 (7.67–14.51) | 326.67 | 1.58 (1.34) | 2.97 (2.62–3.36) | <0.001 | <0.001 |
| oesophageal candidiasis | Infections and infestations | 30 | 70,776 | 8 | 202,557 | 10.73 (4.92–23.41) | 10.73 (4.92–23.40) | 55.72 | 1.48 (0.90) | 2.71 (2.00–3.60) | <0.001 | <0.001 |
| oesophageal haemorrhage | Gastrointestinal disorders | 37 | 70,769 | 8 | 202,557 | 13.24 (6.16–28.43) | 13.23 (6.16–28.41) | 74.39 | 1.55 (1.03) | 2.86 (2.18–3.70) | <0.001 | <0.001 |
| oesophageal perforation | Gastrointestinal disorders | 24 | 70,782 | 3 | 202,562 | 22.89 (6.89–76.03) | 22.89 (6.89–76.00) | 55.82 | 1.60 (0.94) | 2.90 (2.08–3.97) | <0.001 | <0.001 |
| oesophageal ulcer | Gastrointestinal disorders | 76 | 70,730 | 32 | 202,533 | 6.80 (4.50–10.28) | 6.79 (4.50–10.27) | 111.32 | 1.39 (1.04) | 2.60 (2.15–3.12) | <0.001 | <0.001 |
| oesophageal ulcer haemorrhage | Gastrointestinal disorders | 27 | 70,779 | 4 | 202,561 | 19.32 (6.76–55.21) | 19.31 (6.76–55.19) | 60.50 | 1.59 (0.97) | 2.90 (2.11–3.91) | <0.001 | <0.001 |
| oesophagitis haemorrhagic | Gastrointestinal disorders | 21 | 70,785 | 1 | 202,564 | 60.10 (8.08–446.78) | 60.08 (8.08–446.64) | 55.46 | 1.66 (0.94) | 3.00 (2.10–4.19) | <0.001 | <0.001 |
| oesophagitis ulcerative | Gastrointestinal disorders | 26 | 70,780 | 1 | 202,564 | 74.41 (10.10–548.36) | 74.38 (10.09–548.14) | 69.72 | 1.71 (1.06) | 3.13 (2.27–4.23) | <0.001 | <0.001 |
| oral candidiasis | Infections and infestations | 154 | 70,652 | 88 | 202,477 | 5.02 (3.86–6.52) | 5.01 (3.85–6.50) | 179.71 | 1.28 (1.03) | 2.41 (2.11–2.75) | <0.001 | <0.001 |
| orthopnoea | Respiratory, thoracic and mediastinal disorders | 77 | 70,729 | 41 | 202,524 | 5.38 (3.68–7.86) | 5.37 (3.68–7.85) | 95.26 | 1.29 (0.94) | 2.43 (2.01–2.91) | <0.001 | <0.001 |
| orthostatic hypotension | Vascular disorders | 430 | 70,376 | 196 | 202,369 | 6.31 (5.33–7.47) | 6.28 (5.30–7.43) | 598.56 | 1.40 (1.25) | 2.63 (2.43–2.84) | <0.001 | <0.001 |
| orthostatic intolerance | Nervous system disorders | 19 | 70,787 | 1 | 202,564 | 54.37 (7.28–406.16) | 54.36 (7.28–406.04) | 49.76 | 1.63 (0.88) | 2.94 (2.02–4.16) | <0.001 | <0.001 |
| packed red blood cell transfusion | Surgical and medical procedures | 29 | 70,777 | 2 | 202,563 | 41.50 (9.90–173.92) | 41.48 (9.90–173.84) | 73.92 | 1.69 (1.08) | 3.10 (2.28–4.14) | <0.001 | <0.001 |
| pallor | Vascular disorders | 431 | 70,375 | 193 | 202,372 | 6.42 (5.42–7.61) | 6.39 (5.39–7.57) | 607.30 | 1.41 (1.26) | 2.65 (2.45–2.86) | <0.001 | <0.001 |
| peptic ulcer haemorrhage | Gastrointestinal disorders | 30 | 70,776 | 8 | 202,557 | 10.73 (4.92–23.41) | 10.73 (4.92–23.40) | 55.72 | 1.48 (0.90) | 2.71 (2.00–3.60) | <0.001 | <0.001 |
| pericardial disease | Cardiac disorders | 27 | 70,779 | 2 | 202,563 | 38.64 (9.19–162.48) | 38.62 (9.18–162.41) | 68.25 | 1.67 (1.04) | 3.06 (2.23–4.13) | <0.001 | <0.001 |
| pericardial haemorrhage | Cardiac disorders | 70 | 70,736 | 15 | 202,550 | 13.36 (7.65–23.34) | 13.35 (7.64–23.32) | 141.18 | 1.61 (1.23) | 3.00 (2.46–3.63) | <0.001 | <0.001 |
| periorbital haematoma | Injury, poisoning and procedural complications | 33 | 70,773 | 9 | 202,556 | 10.49 (5.02–21.93) | 10.49 (5.02–21.92) | 60.72 | 1.48 (0.93) | 2.72 (2.04–3.57) | <0.001 | <0.001 |
| peripheral venous disease | Vascular disorders | 50 | 70,756 | 11 | 202,554 | 13.01 (6.77–25.00) | 13.00 (6.77–24.98) | 99.93 | 1.58 (1.13) | 2.93 (2.31–3.66) | <0.001 | <0.001 |
| peritoneal haemorrhage | Gastrointestinal disorders | 33 | 70,773 | 4 | 202,561 | 23.61 (8.36–66.65) | 23.60 (8.36–66.62) | 77.23 | 1.65 (1.08) | 3.03 (2.27–3.98) | <0.001 | <0.001 |
| personality disorder | Psychiatric disorders | 192 | 70,614 | 29 | 202,536 | 18.99 (12.85–28.06) | 18.94 (12.82–27.99) | 428.49 | 1.72 (1.49) | 3.28 (2.91–3.69) | <0.001 | <0.001 |
| photophobia | Eye disorders | 160 | 70,646 | 103 | 202,462 | 4.45 (3.47–5.70) | 4.44 (3.47–5.69) | 167.41 | 1.21 (0.98) | 2.31 (2.03–2.62) | <0.001 | <0.001 |
| pneumothorax | Respiratory, thoracic and mediastinal disorders | 122 | 70,684 | 48 | 202,517 | 7.28 (5.21–10.17) | 7.27 (5.21–10.15) | 186.43 | 1.44 (1.16) | 2.70 (2.32–3.12) | <0.001 | <0.001 |
| post procedural haematoma | Injury, poisoning and procedural complications | 53 | 70,753 | 7 | 202,558 | 21.68 (9.85–47.68) | 21.66 (9.85–47.64) | 121.88 | 1.68 (1.24) | 3.14 (2.51–3.91) | <0.001 | <0.001 |
| post procedural haemorrhage | Injury, poisoning and procedural complications | 244 | 70,562 | 21 | 202,544 | 33.35 (21.36–52.09) | 33.24 (21.29–51.90) | 605.23 | 1.81 (1.60) | 3.49 (3.14–3.86) | <0.001 | <0.001 |
| prinzmetal angina | Cardiac disorders | 27 | 70,779 | 6 | 202,559 | 12.88 (5.32–31.19) | 12.87 (5.32–31.18) | 53.77 | 1.51 (0.90) | 2.76 (2.00–3.71) | <0.001 | <0.001 |
| proctitis | Gastrointestinal disorders | 132 | 70,674 | 46 | 202,519 | 8.22 (5.88–11.50) | 8.21 (5.87–11.48) | 216.11 | 1.49 (1.22) | 2.79 (2.41–3.21) | <0.001 | <0.001 |
| product monitoring error | Injury, poisoning and procedural complications | 58 | 70,748 | 8 | 202,557 | 20.76 (9.91–43.47) | 20.74 (9.90–43.44) | 132.13 | 1.68 (1.26) | 3.15 (2.53–3.88) | <0.001 | <0.001 |
| progressive multiple sclerosis | Nervous system disorders | 20 | 70,786 | 1 | 202,564 | 57.23 (7.68–426.47) | 57.22 (7.68–426.34) | 52.61 | 1.65 (0.91) | 2.97 (2.06–4.18) | <0.001 | <0.001 |
| pseudomonal sepsis | Infections and infestations | 22 | 70,784 | 3 | 202,562 | 20.99 (6.28–70.12) | 20.98 (6.28–70.09) | 50.24 | 1.57 (0.88) | 2.84 (2.00–3.94) | <0.001 | <0.001 |
| pulmonary haemorrhage | Respiratory, thoracic and mediastinal disorders | 53 | 70,753 | 21 | 202,544 | 7.22 (4.36–11.98) | 7.22 (4.36–11.97) | 80.62 | 1.40 (0.97) | 2.60 (2.07–3.23) | <0.001 | <0.001 |
| pulmonary valve disease | Cardiac disorders | 21 | 70,785 | 1 | 202,564 | 60.10 (8.08–446.78) | 60.08 (8.08–446.64) | 55.46 | 1.66 (0.94) | 3.00 (2.10–4.19) | <0.001 | <0.001 |
| pulmonary vascular disorder | Respiratory, thoracic and mediastinal disorders | 29 | 70,777 | 3 | 202,562 | 27.67 (8.43–90.82) | 27.65 (8.42–90.78) | 69.86 | 1.65 (1.05) | 3.03 (2.22–4.04) | <0.001 | <0.001 |
| radiculopathy | Nervous system disorders | 162 | 70,644 | 61 | 202,504 | 7.61 (5.67–10.22) | 7.60 (5.66–10.20) | 254.09 | 1.46 (1.22) | 2.75 (2.41–3.12) | <0.001 | <0.001 |
| rectal haemorrhage | Gastrointestinal disorders | 1,390 | 69,416 | 401 | 202,164 | 10.10 (9.03–11.29) | 9.92 (8.88–11.08) | 2511.63 | 1.58 (1.50) | 2.99 (2.86–3.12) | <0.001 | <0.001 |
| relapsing-remitting multiple sclerosis | Nervous system disorders | 56 | 70,750 | 4 | 202,561 | 40.08 (14.53–110.54) | 40.05 (14.52–110.45) | 142.18 | 1.76 (1.32) | 3.32 (2.66–4.10) | <0.001 | <0.001 |
| renal haemorrhage | Renal and urinary disorders | 58 | 70,748 | 14 | 202,551 | 11.86 (6.62–21.26) | 11.85 (6.61–21.24) | 112.09 | 1.57 (1.15) | 2.91 (2.34–3.58) | <0.001 | <0.001 |
| respiratory tract congestion | Respiratory, thoracic and mediastinal disorders | 126 | 70,680 | 62 | 202,503 | 5.82 (4.30–7.89) | 5.81 (4.29–7.88) | 165.74 | 1.34 (1.07) | 2.53 (2.18–2.92) | <0.001 | <0.001 |
| respiratory tract haemorrhage | Respiratory, thoracic and mediastinal disorders | 35 | 70,771 | 0 | 202,565 | 203.22 (12.47–3313.00) | Inf (12.46–3311.26) | 100.14 | 1.80 (1.24) | 3.36 (2.54–4.38) | <0.001 | <0.001 |
| respiratory tract infection | Infections and infestations | 329 | 70,477 | 195 | 202,370 | 4.84 (4.06–5.78) | 4.83 (4.04–5.76) | 372.17 | 1.27 (1.10) | 2.40 (2.20–2.63) | <0.001 | <0.001 |
| retroperitoneal haematoma | Gastrointestinal disorders | 52 | 70,754 | 8 | 202,557 | 18.61 (8.84–39.17) | 18.60 (8.83–39.14) | 115.46 | 1.66 (1.21) | 3.09 (2.45–3.84) | <0.001 | <0.001 |
| retroperitoneal haemorrhage | Gastrointestinal disorders | 45 | 70,761 | 4 | 202,561 | 32.20 (11.58–89.55) | 32.18 (11.58–89.49) | 111.02 | 1.72 (1.23) | 3.21 (2.51–4.06) | <0.001 | <0.001 |
| rhinitis | Infections and infestations | 160 | 70,646 | 103 | 202,462 | 4.45 (3.47–5.70) | 4.44 (3.47–5.69) | 167.41 | 1.21 (0.98) | 2.31 (2.03–2.62) | <0.001 | <0.001 |
| secretion discharge | General disorders and administration site conditions | 159 | 70,647 | 40 | 202,525 | 11.40 (8.06–16.12) | 11.37 (8.04–16.08) | 302.55 | 1.60 (1.35) | 3.01 (2.64–3.42) | <0.001 | <0.001 |
| sedation | Nervous system disorders | 274 | 70,532 | 93 | 202,472 | 8.46 (6.68–10.70) | 8.43 (6.66–10.66) | 455.22 | 1.51 (1.32) | 2.84 (2.58–3.14) | <0.001 | <0.001 |
| sedation complication | Injury, poisoning and procedural complications | 208 | 70,598 | 47 | 202,518 | 12.70 (9.25–17.42) | 12.66 (9.23–17.37) | 412.12 | 1.63 (1.41) | 3.09 (2.76–3.45) | <0.001 | <0.001 |
| sensation of foreign body | General disorders and administration site conditions | 116 | 70,690 | 55 | 202,510 | 6.04 (4.38–8.33) | 6.03 (4.38–8.32) | 156.78 | 1.36 (1.07) | 2.55 (2.18–2.96) | <0.001 | <0.001 |
| shock haemorrhagic | Vascular disorders | 272 | 70,534 | 103 | 202,462 | 7.58 (6.04–9.51) | 7.55 (6.02–9.48) | 425.47 | 1.47 (1.28) | 2.76 (2.50–3.05) | <0.001 | <0.001 |
| sinus congestion | Respiratory, thoracic and mediastinal disorders | 143 | 70,663 | 73 | 202,492 | 5.61 (4.23–7.44) | 5.60 (4.23–7.43) | 182.95 | 1.33 (1.07) | 2.50 (2.18–2.86) | <0.001 | <0.001 |
| skin haemorrhage | Skin and subcutaneous tissue disorders | 206 | 70,600 | 72 | 202,493 | 8.21 (6.27–10.73) | 8.19 (6.26–10.70) | 336.86 | 1.50 (1.28) | 2.81 (2.51–3.15) | <0.001 | <0.001 |
| small for dates baby | Pregnancy, puerperium and perinatal conditions | 17 | 70,789 | 0 | 202,565 | 100.15 (6.02–1665.52) | Inf (6.02–1665.07) | 48.64 | 1.67 (0.87) | 2.98 (2.00–4.31) | <0.001 | <0.001 |
| small intestinal haemorrhage | Gastrointestinal disorders | 80 | 70,726 | 7 | 202,558 | 32.73 (15.12–70.88) | 32.70 (15.10–70.79) | 197.84 | 1.77 (1.40) | 3.35 (2.79–4.01) | <0.001 | <0.001 |
| spinal cord haemorrhage | Nervous system disorders | 17 | 70,789 | 0 | 202,565 | 100.15 (6.02–1665.52) | Inf (6.02–1665.07) | 48.64 | 1.67 (0.87) | 2.98 (2.00–4.31) | <0.001 | <0.001 |
| spontaneous haematoma | Blood and lymphatic system disorders | 38 | 70,768 | 5 | 202,560 | 21.75 (8.56–55.27) | 21.74 (8.56–55.24) | 87.45 | 1.65 (1.13) | 3.05 (2.34–3.94) | <0.001 | <0.001 |
| spontaneous haemorrhage | Blood and lymphatic system disorders | 22 | 70,784 | 2 | 202,563 | 31.48 (7.40–133.87) | 31.47 (7.40–133.83) | 54.09 | 1.62 (0.93) | 2.93 (2.06–4.07) | <0.001 | <0.001 |
| stenosis | General disorders and administration site conditions | 68 | 70,738 | 35 | 202,530 | 5.56 (3.70–8.36) | 5.56 (3.70–8.36) | 86.41 | 1.30 (0.93) | 2.44 (2.00–2.96) | <0.001 | <0.001 |
| steroid dependence | General disorders and administration site conditions | 129 | 70,677 | 1 | 202,564 | 369.72 (51.68–2644.80) | 369.05 (51.59–2639.89) | 364.40 | 1.90 (1.60) | 3.68 (3.18–4.24) | <0.001 | <0.001 |
| subcutaneous haematoma | Injury, poisoning and procedural complications | 44 | 70,762 | 6 | 202,559 | 20.99 (8.95–49.26) | 20.98 (8.94–49.23) | 100.48 | 1.66 (1.17) | 3.09 (2.40–3.91) | <0.001 | <0.001 |
| subdural haematoma | Injury, poisoning and procedural complications | 464 | 70,342 | 170 | 202,395 | 7.85 (6.59–9.36) | 7.81 (6.55–9.31) | 740.31 | 1.49 (1.35) | 2.80 (2.59–3.02) | <0.001 | <0.001 |
| subdural haemorrhage | Injury, poisoning and procedural complications | 83 | 70,723 | 17 | 202,548 | 13.98 (8.30–23.56) | 13.97 (8.29–23.53) | 169.94 | 1.63 (1.28) | 3.05 (2.54–3.64) | <0.001 | <0.001 |
| systemic infection | Infections and infestations | 77 | 70,729 | 1 | 202,564 | 220.52 (30.67–1585.59) | 220.29 (30.64–1583.81) | 215.55 | 1.86 (1.48) | 3.57 (2.95–4.28) | <0.001 | <0.001 |
| thalamus haemorrhage | Nervous system disorders | 59 | 70,747 | 2 | 202,563 | 84.46 (20.64–345.70) | 84.39 (20.62–345.40) | 159.45 | 1.81 (1.38) | 3.44 (2.77–4.23) | <0.001 | <0.001 |
| thrombosis in device | Product issues | 62 | 70,744 | 9 | 202,556 | 19.72 (9.80–39.69) | 19.71 (9.80–39.65) | 139.61 | 1.68 (1.27) | 3.15 (2.55–3.85) | <0.001 | <0.001 |
| transfusion | Surgical and medical procedures | 65 | 70,741 | 15 | 202,550 | 12.41 (7.08–21.75) | 12.40 (7.07–21.73) | 127.73 | 1.58 (1.19) | 2.95 (2.40–3.60) | <0.001 | <0.001 |
| traumatic haemorrhage | Injury, poisoning and procedural complications | 44 | 70,762 | 1 | 202,564 | 125.95 (17.35–914.24) | 125.88 (17.34–913.64) | 121.15 | 1.80 (1.30) | 3.38 (2.64–4.29) | <0.001 | <0.001 |
| truncus arteriosus persistent | Congenital, familial and genetic disorders | 67 | 70,739 | 0 | 202,565 | 386.58 (23.93–6244.48) | Inf (23.91–6238.38) | 191.72 | 1.87 (1.46) | 3.58 (2.92–4.35) | <0.001 | <0.001 |
| type v hyperlipidaemia | Congenital, familial and genetic disorders | 35 | 70,771 | 11 | 202,554 | 9.11 (4.62–17.93) | 9.10 (4.62–17.92) | 60.38 | 1.45 (0.92) | 2.66 (2.02–3.47) | <0.001 | <0.001 |
| ulcer haemorrhage | General disorders and administration site conditions | 87 | 70,719 | 45 | 202,520 | 5.54 (3.86–7.94) | 5.53 (3.86–7.93) | 110.14 | 1.31 (0.98) | 2.46 (2.06–2.92) | <0.001 | <0.001 |
| upper gastrointestinal haemorrhage | Gastrointestinal disorders | 1,035 | 69,771 | 91 | 202,474 | 33.01 (26.63–40.90) | 32.54 (26.27–40.31) | 2567.53 | 1.82 (1.72) | 3.53 (3.36–3.71) | <0.001 | <0.001 |
| urinary bladder haemorrhage | Renal and urinary disorders | 50 | 70,756 | 7 | 202,558 | 20.45 (9.27–45.10) | 20.43 (9.27–45.07) | 113.52 | 1.67 (1.21) | 3.11 (2.46–3.89) | <0.001 | <0.001 |
| urinary tract disorder | Renal and urinary disorders | 195 | 70,611 | 48 | 202,517 | 11.65 (8.50–15.98) | 11.62 (8.48–15.94) | 374.28 | 1.61 (1.38) | 3.04 (2.70–3.41) | <0.001 | <0.001 |
| urogenital haemorrhage | Renal and urinary disorders | 39 | 70,767 | 2 | 202,563 | 55.82 (13.48–231.16) | 55.79 (13.47–231.03) | 102.37 | 1.75 (1.22) | 3.26 (2.51–4.19) | <0.001 | <0.001 |
| vaginal discharge | Reproductive system and breast disorders | 134 | 70,672 | 46 | 202,519 | 8.35 (5.97–11.67) | 8.33 (5.96–11.65) | 221.15 | 1.49 (1.22) | 2.80 (2.43–3.21) | <0.001 | <0.001 |
| vaginal flatulence | Reproductive system and breast disorders | 123 | 70,683 | 29 | 202,536 | 12.15 (8.11–18.22) | 12.13 (8.10–18.18) | 239.88 | 1.61 (1.32) | 3.02 (2.61–3.49) | <0.001 | <0.001 |
| vascular pseudoaneurysm | Injury, poisoning and procedural complications | 67 | 70,739 | 15 | 202,550 | 12.79 (7.31–22.39) | 12.78 (7.30–22.37) | 133.10 | 1.59 (1.20) | 2.97 (2.43–3.61) | <0.001 | <0.001 |
| vascular stent stenosis | General disorders and administration site conditions | 52 | 70,754 | 21 | 202,544 | 7.09 (4.27–11.77) | 7.08 (4.27–11.76) | 78.18 | 1.39 (0.96) | 2.59 (2.05–3.22) | <0.001 | <0.001 |
| vascular stent thrombosis | General disorders and administration site conditions | 88 | 70,718 | 5 | 202,560 | 50.41 (20.47–124.14) | 50.35 (20.45–123.97) | 228.93 | 1.81 (1.46) | 3.46 (2.90–4.10) | <0.001 | <0.001 |
| ventricular arrhythmia | Cardiac disorders | 78 | 70,728 | 36 | 202,529 | 6.20 (4.18–9.21) | 6.20 (4.18–9.20) | 107.43 | 1.36 (1.01) | 2.54 (2.11–3.04) | <0.001 | <0.001 |
| ventricular septal defect | Congenital, familial and genetic disorders | 84 | 70,722 | 22 | 202,543 | 10.94 (6.84–17.49) | 10.92 (6.83–17.47) | 157.22 | 1.56 (1.22) | 2.92 (2.44–3.48) | <0.001 | <0.001 |
| vital functions abnormal | Investigations | 21 | 70,785 | 2 | 202,563 | 30.05 (7.05–128.15) | 30.04 (7.04–128.11) | 51.27 | 1.61 (0.90) | 2.90 (2.03–4.05) | <0.001 | <0.001 |
| vitreous haemorrhage | Eye disorders | 50 | 70,756 | 21 | 202,544 | 6.82 (4.09–11.35) | 6.81 (4.09–11.34) | 73.35 | 1.37 (0.94) | 2.55 (2.02–3.19) | <0.001 | <0.001 |
| wound dehiscence | Injury, poisoning and procedural complications | 47 | 70,759 | 15 | 202,550 | 8.97 (5.02–16.04) | 8.96 (5.01–16.03) | 80.47 | 1.47 (1.01) | 2.72 (2.14–3.42) | <0.001 | <0.001 |
| wound infection pseudomonas | Infections and infestations | 33 | 70,773 | 0 | 202,565 | 191.77 (11.75–3129.89) | Inf (11.74–3128.33) | 94.42 | 1.79 (1.21) | 3.34 (2.50–4.38) | <0.001 | <0.001 |

**Note:** PT: Preferred Term; SOC: System Organ Class; a: Co-medicated reports with the PT; b: Co-medicated reports without the PT; c: Comparator reports with the PT; d: Comparator reports without the PT; ROR: Reporting Odds Ratio; PRR: Proportional Reporting Ratio; IC: Information Component; IC025: Lower 95% credibility limit of IC; EBGM: Empirical Bayes Geometric Mean; EB05: Lower 95% credibility limit of EBGM; EB95: Upper 95% credibility limit of EBGM; BH-FDR: Benjamini–Hochberg false discovery rate.

**Table S2.** Disproportionality signals: Co-medicated vs Statins without aspirin.
