# Supplementary material for: Disproportionality analysis of adverse event reporting with aspirin, statins, and their co-exposure in FAERS, 2004–2025
Source: Front Med (Lausanne). 2026 Oct 1;13:1923188. doi: 10.3389/fmed.2026.1923188 (PMC13669111; doi:10.3389/fmed.2026.1923188)
Supplement: Supplementary file 3 [file Table3.docx]

| **PT** | **Primary SOC** | **Co-medicated reports, n** | **ROR \| Aspirin comparator** | **PRR \| Aspirin comparator** | **IC (IC025) \| Aspirin comparator** | **EBGM (EB05-EB95) \| Aspirin comparator** | **BH-FDR \| Aspirin comparator** | **ROR \| Statin comparator** | **PRR \| Statin comparator** | **IC (IC025) \| Statin comparator** | **EBGM (EB05-EB95) \| Statin comparator** | **BH-FDR \| Statin comparator** | **Minimum EB05** |
| --- | --- | --- | --- | --- | --- | --- | --- | --- | --- | --- | --- | --- | --- |
| sedation complication | Injury, poisoning and procedural complications | 208 | 198.10 (49.21-797.43) | 197.52 (49.07-795.05) | 1.50 (1.27) | 2.76 (2.46-3.08) | <0.001 | 12.70 (9.25-17.42) | 12.66 (9.23-17.37) | 1.63 (1.41) | 3.09 (2.76-3.45) | <0.001 | 2.46 |
| adjustment disorder | Psychiatric disorders | 198 | 37.71 (19.98-71.18) | 37.61 (19.92-70.98) | 1.44 (1.21) | 2.66 (2.36-2.98) | <0.001 | 40.57 (23.59-69.77) | 40.46 (23.53-69.57) | 1.82 (1.59) | 3.52 (3.12-3.95) | <0.001 | 2.36 |
| catarrh | Respiratory, thoracic and mediastinal disorders | 121 | 230.20 (32.16-1647.58) | 229.81 (32.11-1644.71) | 1.49 (1.19) | 2.69 (2.32-3.11) | <0.001 | 17.34 (10.80-27.83) | 17.31 (10.78-27.78) | 1.69 (1.40) | 3.20 (2.75-3.70) | <0.001 | 2.32 |
| affect lability | Psychiatric disorders | 200 | 23.81 (14.30-39.61) | 23.74 (14.27-39.50) | 1.41 (1.17) | 2.59 (2.30-2.90) | <0.001 | 11.95 (8.72-16.38) | 11.92 (8.70-16.33) | 1.62 (1.39) | 3.05 (2.71-3.42) | <0.001 | 2.30 |
| radiculopathy | Nervous system disorders | 162 | 30.84 (16.28-58.41) | 30.77 (16.25-58.27) | 1.43 (1.17) | 2.61 (2.30-2.96) | <0.001 | 7.61 (5.67-10.22) | 7.60 (5.66-10.20) | 1.46 (1.22) | 2.75 (2.41-3.12) | <0.001 | 2.30 |
| vaginal flatulence | Reproductive system and breast disorders | 123 | 78.00 (24.81-245.21) | 77.87 (24.77-244.77) | 1.47 (1.17) | 2.66 (2.30-3.07) | <0.001 | 12.15 (8.11-18.22) | 12.13 (8.10-18.18) | 1.61 (1.32) | 3.02 (2.61-3.49) | <0.001 | 2.30 |
| amaurosis fugax | Eye disorders | 160 | 27.69 (15.03-51.01) | 27.63 (15.00-50.89) | 1.42 (1.16) | 2.59 (2.28-2.94) | <0.001 | 28.67 (17.15-47.94) | 28.61 (17.11-47.82) | 1.78 (1.52) | 3.41 (2.99-3.87) | <0.001 | 2.28 |
| burns second degree | Injury, poisoning and procedural complications | 125 | 59.46 (21.97-160.91) | 59.35 (21.93-160.61) | 1.46 (1.16) | 2.64 (2.28-3.05) | <0.001 | 23.88 (13.98-40.80) | 23.84 (13.96-40.73) | 1.75 (1.46) | 3.32 (2.86-3.84) | <0.001 | 2.28 |
| personality disorder | Psychiatric disorders | 192 | 21.51 (13.10-35.32) | 21.45 (13.06-35.22) | 1.39 (1.16) | 2.57 (2.28-2.88) | <0.001 | 18.99 (12.85-28.06) | 18.94 (12.82-27.99) | 1.72 (1.49) | 3.28 (2.91-3.69) | <0.001 | 2.28 |
| nasal oedema | Respiratory, thoracic and mediastinal disorders | 126 | 47.94 (19.61-117.20) | 47.86 (19.58-116.98) | 1.45 (1.15) | 2.63 (2.27-3.02) | <0.001 | 13.37 (8.82-20.27) | 13.35 (8.81-20.23) | 1.63 (1.35) | 3.08 (2.65-3.55) | <0.001 | 2.27 |
| fractional exhaled nitric oxide increased | Investigations | 94 | 359.44 (22.32-5789.11) | Inf (22.29-5781.23) | 1.49 (1.15) | 2.67 (2.25-3.14) | <0.001 | 541.42 (33.62-8720.07) | Inf (33.57-8708.23) | 1.89 (1.55) | 3.65 (3.08-4.31) | <0.001 | 2.25 |
| gingival pain | Gastrointestinal disorders | 139 | 26.45 (13.92-50.25) | 26.40 (13.90-50.15) | 1.41 (1.13) | 2.57 (2.24-2.94) | <0.001 | 6.42 (4.76-8.67) | 6.41 (4.76-8.65) | 1.39 (1.13) | 2.61 (2.27-2.99) | <0.001 | 2.24 |
| steroid dependence | General disorders and administration site conditions | 129 | 27.27 (13.87-53.60) | 27.22 (13.85-53.50) | 1.41 (1.12) | 2.56 (2.22-2.95) | <0.001 | 369.72 (51.68-2644.80) | 369.05 (51.59-2639.89) | 1.90 (1.60) | 3.68 (3.18-4.24) | <0.001 | 2.22 |
| congenital hiatus hernia | Congenital, familial and genetic disorders | 83 | 317.55 (19.70-5119.38) | Inf (19.67-5113.21) | 1.49 (1.12) | 2.64 (2.21-3.13) | <0.001 | 47.54 (19.28-117.24) | 47.49 (19.26-117.10) | 1.80 (1.44) | 3.44 (2.86-4.10) | <0.001 | 2.21 |
| malignant melanoma in situ | Neoplasms benign, malignant and unspecified (incl cysts and polyps) | 82 | 313.74 (19.46-5058.50) | Inf (19.44-5052.48) | 1.49 (1.12) | 2.64 (2.21-3.13) | <0.001 | 46.97 (19.04-115.87) | 46.92 (19.02-115.73) | 1.80 (1.44) | 3.43 (2.86-4.10) | <0.001 | 2.21 |
| ocular discomfort | Eye disorders | 177 | 16.85 (10.61-26.75) | 16.81 (10.59-26.69) | 1.36 (1.12) | 2.51 (2.21-2.83) | <0.001 | 10.57 (7.68-14.55) | 10.55 (7.67-14.51) | 1.58 (1.34) | 2.97 (2.62-3.36) | <0.001 | 2.21 |
| vaginal discharge | Reproductive system and breast disorders | 134 | 23.18 (12.53-42.86) | 23.14 (12.51-42.78) | 1.39 (1.11) | 2.54 (2.21-2.92) | <0.001 | 8.35 (5.97-11.67) | 8.33 (5.96-11.65) | 1.49 (1.22) | 2.80 (2.43-3.21) | <0.001 | 2.21 |
| carotid artery thrombosis | Nervous system disorders | 177 | 14.65 (9.49-22.62) | 14.62 (9.47-22.57) | 1.34 (1.09) | 2.47 (2.18-2.79) | <0.001 | 46.15 (25.10-84.85) | 46.03 (25.04-84.64) | 1.83 (1.58) | 3.54 (3.12-3.99) | <0.001 | 2.18 |
| neurologic neglect syndrome | Nervous system disorders | 173 | 14.97 (9.60-23.33) | 14.94 (9.58-23.27) | 1.34 (1.09) | 2.47 (2.18-2.79) | <0.001 | 70.87 (33.29-150.90) | 70.70 (33.21-150.52) | 1.86 (1.61) | 3.60 (3.18-4.07) | <0.001 | 2.18 |
| sedation | Nervous system disorders | 274 | 10.66 (7.86-14.45) | 10.62 (7.84-14.39) | 1.29 (1.09) | 2.40 (2.18-2.65) | <0.001 | 8.46 (6.68-10.70) | 8.43 (6.66-10.66) | 1.51 (1.32) | 2.84 (2.58-3.14) | <0.001 | 2.18 |
| blood calcium decreased | Investigations | 265 | 10.75 (7.88-14.66) | 10.71 (7.85-14.60) | 1.29 (1.09) | 2.40 (2.17-2.65) | <0.001 | 5.43 (4.43-6.67) | 5.42 (4.41-6.64) | 1.32 (1.14) | 2.50 (2.25-2.76) | <0.001 | 2.17 |
| proctitis | Gastrointestinal disorders | 132 | 19.32 (10.93-34.15) | 19.28 (10.91-34.09) | 1.37 (1.09) | 2.50 (2.17-2.87) | <0.001 | 8.22 (5.88-11.50) | 8.21 (5.87-11.48) | 1.49 (1.22) | 2.79 (2.41-3.21) | <0.001 | 2.17 |
| respiratory tract infection | Infections and infestations | 329 | 9.51 (7.30-12.39) | 9.47 (7.27-12.33) | 1.26 (1.08) | 2.37 (2.17-2.59) | <0.001 | 4.84 (4.06-5.78) | 4.83 (4.04-5.76) | 1.27 (1.10) | 2.40 (2.20-2.63) | <0.001 | 2.17 |
| urinary tract disorder | Renal and urinary disorders | 195 | 12.80 (8.67-18.91) | 12.77 (8.65-18.86) | 1.32 (1.09) | 2.44 (2.17-2.74) | <0.001 | 11.65 (8.50-15.98) | 11.62 (8.48-15.94) | 1.61 (1.38) | 3.04 (2.70-3.41) | <0.001 | 2.17 |
| female genital tract fistula | Reproductive system and breast disorders | 130 | 17.67 (10.18-30.66) | 17.64 (10.16-30.61) | 1.36 (1.07) | 2.48 (2.15-2.86) | <0.001 | 11.64 (7.91-17.14) | 11.62 (7.89-17.11) | 1.60 (1.32) | 3.01 (2.60-3.46) | <0.001 | 2.15 |
| frequent bowel movements | Gastrointestinal disorders | 303 | 10.32 (7.76-13.72) | 10.28 (7.73-13.66) | 1.28 (1.09) | 2.40 (2.18-2.63) | <0.001 | 4.60 (3.84-5.52) | 4.59 (3.83-5.50) | 1.24 (1.07) | 2.36 (2.14-2.59) | <0.001 | 2.14 |
| orthostatic hypotension | Vascular disorders | 430 | 7.97 (6.43-9.89) | 7.93 (6.40-9.83) | 1.22 (1.06) | 2.31 (2.13-2.49) | <0.001 | 6.31 (5.33-7.47) | 6.28 (5.30-7.43) | 1.40 (1.25) | 2.63 (2.43-2.84) | <0.001 | 2.13 |
| truncus arteriosus persistent | Congenital, familial and genetic disorders | 67 | 256.64 (15.89-4145.60) | Inf (15.87-4141.54) | 1.48 (1.07) | 2.59 (2.13-3.12) | <0.001 | 386.58 (23.93-6244.48) | Inf (23.91-6238.38) | 1.87 (1.46) | 3.58 (2.92-4.35) | <0.001 | 2.13 |
| oral candidiasis | Infections and infestations | 154 | 12.74 (8.22-19.75) | 12.72 (8.21-19.71) | 1.31 (1.05) | 2.42 (2.12-2.75) | <0.001 | 5.02 (3.86-6.52) | 5.01 (3.85-6.50) | 1.28 (1.03) | 2.41 (2.11-2.75) | <0.001 | 2.11 |
| impaired quality of life | Social circumstances | 171 | 10.85 (7.36-15.99) | 10.83 (7.35-15.96) | 1.28 (1.04) | 2.38 (2.10-2.69) | <0.001 | 9.43 (6.91-12.86) | 9.41 (6.90-12.83) | 1.54 (1.30) | 2.90 (2.55-3.28) | <0.001 | 2.10 |
| brain natriuretic peptide increased | Investigations | 140 | 12.11 (7.72-18.98) | 12.09 (7.71-18.94) | 1.30 (1.03) | 2.39 (2.09-2.74) | <0.001 | 7.43 (5.43-10.17) | 7.42 (5.42-10.15) | 1.45 (1.19) | 2.72 (2.36-3.11) | <0.001 | 2.09 |
| gait spastic | Nervous system disorders | 59 | 226.20 (13.98-3658.88) | Inf (13.97-3655.71) | 1.47 (1.03) | 2.56 (2.08-3.12) | <0.001 | 7.34 (4.54-11.89) | 7.34 (4.53-11.88) | 1.41 (1.01) | 2.63 (2.12-3.23) | <0.001 | 2.08 |
| colitis ulcerative | Gastrointestinal disorders | 303 | 7.60 (5.91-9.77) | 7.57 (5.89-9.74) | 1.20 (1.02) | 2.28 (2.07-2.50) | <0.001 | 5.21 (4.31-6.29) | 5.19 (4.30-6.27) | 1.30 (1.13) | 2.47 (2.24-2.71) | <0.001 | 2.07 |
| creatinine renal clearance decreased | Investigations | 256 | 7.74 (5.88-10.20) | 7.72 (5.86-10.17) | 1.21 (1.01) | 2.28 (2.05-2.52) | <0.001 | 8.96 (6.99-11.49) | 8.93 (6.97-11.45) | 1.53 (1.34) | 2.88 (2.60-3.19) | <0.001 | 2.05 |
| needle fatigue | Injury, poisoning and procedural complications | 56 | 214.79 (13.27-3476.40) | Inf (13.26-3473.53) | 1.46 (1.02) | 2.54 (2.05-3.12) | <0.001 | 80.17 (19.56-328.52) | 80.10 (19.55-328.24) | 1.81 (1.36) | 3.42 (2.74-4.23) | <0.001 | 2.05 |
| muscle spasticity | Nervous system disorders | 258 | 7.34 (5.61-9.60) | 7.31 (5.59-9.57) | 1.19 (0.99) | 2.25 (2.03-2.49) | <0.001 | 7.71 (6.10-9.75) | 7.69 (6.08-9.72) | 1.48 (1.28) | 2.78 (2.51-3.07) | <0.001 | 2.03 |
| relapsing-remitting multiple sclerosis | Nervous system disorders | 56 | 106.44 (14.73-768.96) | 106.36 (14.72-768.33) | 1.44 (1.00) | 2.51 (2.02-3.08) | <0.001 | 40.08 (14.53-110.54) | 40.05 (14.52-110.45) | 1.76 (1.32) | 3.32 (2.66-4.10) | <0.001 | 2.02 |
| secretion discharge | General disorders and administration site conditions | 159 | 8.90 (6.14-12.89) | 8.88 (6.13-12.86) | 1.24 (0.98) | 2.30 (2.02-2.61) | <0.001 | 11.40 (8.06-16.12) | 11.37 (8.04-16.08) | 1.60 (1.35) | 3.01 (2.64-3.42) | <0.001 | 2.02 |
| congenital cardiovascular anomaly | Congenital, familial and genetic disorders | 58 | 55.12 (13.46-225.70) | 55.08 (13.45-225.51) | 1.42 (0.99) | 2.48 (2.01-3.04) | <0.001 | 33.21 (13.32-82.81) | 33.19 (13.31-82.73) | 1.75 (1.32) | 3.28 (2.64-4.04) | <0.001 | 2.01 |
| micturition urgency | Renal and urinary disorders | 190 | 30.15 (16.82-54.04) | 30.07 (16.78-53.89) | 1.43 (1.19) | 2.62 (2.33-2.95) | <0.001 | 4.16 (3.33-5.20) | 4.15 (3.32-5.18) | 1.18 (0.96) | 2.25 (2.00-2.53) | <0.001 | 2.00 |
| obstructive sleep apnoea syndrome | Respiratory, thoracic and mediastinal disorders | 92 | 87.48 (21.55-355.08) | 87.37 (21.53-354.60) | 1.46 (1.11) | 2.61 (2.21-3.08) | <0.001 | 4.97 (3.55-6.97) | 4.97 (3.54-6.96) | 1.26 (0.94) | 2.38 (2.00-2.81) | <0.001 | 2.00 |
| sinus congestion | Respiratory, thoracic and mediastinal disorders | 143 | 8.78 (5.95-12.94) | 8.76 (5.94-12.92) | 1.23 (0.96) | 2.29 (2.00-2.61) | <0.001 | 5.61 (4.23-7.44) | 5.60 (4.23-7.43) | 1.33 (1.07) | 2.50 (2.18-2.86) | <0.001 | 2.00 |

**Note:** PT: Preferred Term; SOC: System Organ Class; ROR: Reporting Odds Ratio; PRR: Proportional Reporting Ratio; IC: Information Component; IC025: Lower 95% credibility limit of IC; EBGM: Empirical Bayes Geometric Mean; EB05: Lower 95% credibility limit of EBGM; EB95: Upper 95% credibility limit of EBGM; BH-FDR: Benjamini-Hochberg false discovery rate; Aspirin comparator: Co-medicated vs Aspirin without statins; Statin comparator: Co-medicated vs Statins without aspirin; Minimum EB05: the lower of the two EB05 values for the same PT. These 42 PTs are common candidate pharmacovigilance signals and should not be interpreted as confirmed drug-drug interactions.

**Table S3.** Full disproportionality results for the 42 common candidate PT signals.
