# Supplementary material for: Disproportionality analysis of adverse event reporting with aspirin, statins, and their co-exposure in FAERS, 2004–2025
Source: Front Med (Lausanne). 2026 Oct 1;13:1923188. doi: 10.3389/fmed.2026.1923188 (PMC13669111; doi:10.3389/fmed.2026.1923188)
Supplement: Supplementary file 4 [file Table4.docx]

| **PT** | **Primary SOC** | **PT reports, n** | **Estimability** | **Adjusted OR A** | **Adjusted OR B** | **Adjusted OR AB** | **IR (95% CI)** | **BH-FDR (IR)** | **OR-based RERI (95% CI)** | **BH-FDR (RERI)** | **Additive positive** | **Multiplicative positive** | **Both-scale positive** |
| --- | --- | --- | --- | --- | --- | --- | --- | --- | --- | --- | --- | --- | --- |
| adjustment disorder | Psychiatric disorders | 1,123 | Reliably estimable | 1.88 | 1.56 | 17.07 | 5.83 (3.73-9.11) | <0.001 | 14.64 (12.03-17.25) | <0.001 | Yes | Yes | Yes |
| affect lability | Psychiatric disorders | 8,974 | Reliably estimable | 0.64 | 0.96 | 2.35 | 3.81 (3.00-4.85) | <0.001 | 1.75 (1.43-2.07) | <0.001 | Yes | Yes | Yes |
| amaurosis fugax | Eye disorders | 884 | Reliably estimable | 2.82 | 2.71 | 16.51 | 2.16 (1.43-3.27) | <0.001 | 11.98 (9.17-14.78) | <0.001 | Yes | Yes | Yes |
| blood calcium decreased | Investigations | 10,226 | Reliably estimable | 1.56 | 1.54 | 2.94 | 1.22 (1.05-1.41) | 0.012 | 0.83 (0.53-1.13) | <0.001 | Yes | Yes | Yes |
| brain natriuretic peptide increased | Investigations | 2,796 | Reliably estimable | 2.74 | 2.54 | 5.12 | 0.74 (0.59-0.91) | 0.008 | 0.84 (0.10-1.58) | 0.031 | Yes | No | No |
| burns second degree | Injury, poisoning and procedural complications | 1,625 | Reliably estimable | 1.00 | 1.14 | 6.22 | 5.46 (3.48-8.55) | <0.001 | 5.08 (3.95-6.21) | <0.001 | Yes | Yes | Yes |
| carotid artery thrombosis | Nervous system disorders | 689 | Reliably estimable | 5.00 | 2.03 | 25.90 | 2.56 (1.62-4.03) | <0.001 | 19.87 (15.40-24.35) | <0.001 | Yes | Yes | Yes |
| catarrh | Respiratory, thoracic and mediastinal disorders | 785 | Reliably estimable | 0.54 | 2.69 | 14.36 | 9.91 (4.42-22.24) | <0.001 | 12.14 (9.33-14.95) | <0.001 | Yes | Yes | Yes |
| colitis ulcerative | Gastrointestinal disorders | 37,949 | Reliably estimable | 0.69 | 0.92 | 1.25 | 1.96 (1.72-2.22) | <0.001 | 0.64 (0.51-0.76) | <0.001 | Yes | Yes | Yes |
| congenital cardiovascular anomaly | Congenital, familial and genetic disorders | 798 | Reliably estimable | 0.16 | 0.23 | 10.17 | 282.46 (84.58-943.28) | <0.001 | 10.79 (8.00-13.57) | <0.001 | Yes | Yes | Yes |
| congenital hiatus hernia | Congenital, familial and genetic disorders | 103 | Not reliably estimable | 0.00 | 7.61 | 226.72 | 145024310.50 (0.00-Inf) |  | 220.12 (84.79-355.44) |  |  |  |  |
| creatinine renal clearance decreased | Investigations | 3,586 | Reliably estimable | 1.88 | 1.68 | 4.18 | 1.32 (1.08-1.62) | 0.010 | 1.61 (1.09-2.13) | <0.001 | Yes | Yes | Yes |
| female genital tract fistula | Reproductive system and breast disorders | 1,492 | Reliably estimable | 1.37 | 1.52 | 7.23 | 3.46 (2.28-5.26) | <0.001 | 5.33 (3.99-6.68) | <0.001 | Yes | Yes | Yes |
| fractional exhaled nitric oxide increased | Investigations | 130 | Not reliably estimable | 0.00 | 1.07 | 211.46 | 611553852.35 (0.00-Inf) |  | 211.39 (120.62-302.15) |  |  |  |  |
| frequent bowel movements | Gastrointestinal disorders | 23,452 | Reliably estimable | 1.10 | 1.26 | 1.84 | 1.33 (1.17-1.51) | <0.001 | 0.48 (0.30-0.66) | <0.001 | Yes | Yes | Yes |
| gait spastic | Nervous system disorders | 308 | Reliably estimable | 2.41 | 6.07 | 22.54 | 1.54 (0.73-3.25) | 0.288 | 15.05 (8.64-21.47) | <0.001 | Yes | No | No |
| gingival pain | Gastrointestinal disorders | 7,292 | Reliably estimable | 1.31 | 1.44 | 2.40 | 1.27 (1.04-1.53) | 0.019 | 0.64 (0.31-0.98) | <0.001 | Yes | Yes | Yes |
| impaired quality of life | Social circumstances | 12,824 | Reliably estimable | 0.77 | 2.08 | 2.48 | 1.55 (1.28-1.86) | <0.001 | 0.63 (0.32-0.95) | <0.001 | Yes | Yes | Yes |
| malignant melanoma in situ | Neoplasms benign, malignant and unspecified (incl cysts and polyps) | 1,250 | Reliably estimable | 1.58 | 1.56 | 4.72 | 1.91 (1.31-2.78) | 0.001 | 2.58 (1.59-3.56) | <0.001 | Yes | Yes | Yes |
| micturition urgency | Renal and urinary disorders | 10,171 | Reliably estimable | 1.54 | 1.99 | 2.87 | 0.93 (0.80-1.09) | 0.399 | 0.33 (0.01-0.66) | 0.048 | Yes | No | No |
| muscle spasticity | Nervous system disorders | 11,603 | Reliably estimable | 0.84 | 0.83 | 2.23 | 3.20 (2.62-3.90) | <0.001 | 1.56 (1.30-1.82) | <0.001 | Yes | Yes | Yes |
| nasal oedema | Respiratory, thoracic and mediastinal disorders | 1,584 | Reliably estimable | 1.48 | 1.34 | 6.15 | 3.10 (2.09-4.60) | <0.001 | 4.33 (3.20-5.46) | <0.001 | Yes | Yes | Yes |
| needle fatigue | Injury, poisoning and procedural complications | 388 | Reliably estimable | 0.38 | 0.92 | 17.57 | 50.42 (10.44-243.47) | <0.001 | 17.28 (12.27-22.28) | <0.001 | Yes | Yes | Yes |
| neurologic neglect syndrome | Nervous system disorders | 418 | Reliably estimable | 7.10 | 3.81 | 64.60 | 2.39 (1.35-4.21) | 0.004 | 54.69 (41.22-68.16) | <0.001 | Yes | Yes | Yes |
| obstructive sleep apnoea syndrome | Respiratory, thoracic and mediastinal disorders | 1,003 | Reliably estimable | 4.89 | 6.81 | 22.17 | 0.67 (0.45-0.98) | 0.045 | 11.47 (7.43-15.51) | <0.001 | Yes | No | No |
| ocular discomfort | Eye disorders | 8,852 | Reliably estimable | 1.18 | 1.67 | 2.72 | 1.39 (1.13-1.69) | 0.002 | 0.88 (0.51-1.24) | <0.001 | Yes | Yes | Yes |
| oral candidiasis | Infections and infestations | 11,451 | Reliably estimable | 1.41 | 1.55 | 2.08 | 0.95 (0.82-1.11) | 0.547 | 0.12 (-0.14-0.38) | 0.372 | No | No | No |
| orthostatic hypotension | Vascular disorders | 15,783 | Reliably estimable | 2.07 | 2.29 | 3.57 | 0.75 (0.68-0.83) | <0.001 | 0.21 (-0.04-0.46) | 0.101 | No | No | No |
| personality disorder | Psychiatric disorders | 2,364 | Reliably estimable | 1.51 | 1.35 | 6.61 | 3.25 (2.34-4.51) | <0.001 | 4.75 (3.76-5.75) | <0.001 | Yes | Yes | Yes |
| proctitis | Gastrointestinal disorders | 2,480 | Reliably estimable | 1.30 | 1.60 | 4.21 | 2.02 (1.48-2.77) | <0.001 | 2.31 (1.58-3.04) | <0.001 | Yes | Yes | Yes |
| radiculopathy | Nervous system disorders | 2,434 | Reliably estimable | 2.23 | 2.47 | 7.90 | 1.44 (1.10-1.87) | 0.009 | 4.21 (3.13-5.29) | <0.001 | Yes | Yes | Yes |
| relapsing-remitting multiple sclerosis | Nervous system disorders | 762 | Reliably estimable | 1.03 | 0.93 | 7.53 | 7.84 (3.72-16.51) | <0.001 | 6.57 (4.52-8.62) | <0.001 | Yes | Yes | Yes |
| respiratory tract infection | Infections and infestations | 22,968 | Reliably estimable | 1.43 | 1.40 | 2.19 | 1.10 (0.98-1.23) | 0.125 | 0.36 (0.17-0.56) | <0.001 | Yes | No | No |
| secretion discharge | General disorders and administration site conditions | 10,555 | Reliably estimable | 1.36 | 1.39 | 2.07 | 1.09 (0.92-1.30) | 0.359 | 0.31 (0.03-0.60) | 0.036 | Yes | No | No |
| sedation | Nervous system disorders | 21,882 | Reliably estimable | 0.72 | 0.80 | 1.31 | 2.29 (1.97-2.65) | <0.001 | 0.79 (0.65-0.94) | <0.001 | Yes | Yes | Yes |
| sedation complication | Injury, poisoning and procedural complications | 1,866 | Reliably estimable | 0.97 | 2.48 | 9.04 | 3.76 (2.52-5.60) | <0.001 | 6.59 (5.28-7.90) | <0.001 | Yes | Yes | Yes |
| sinus congestion | Respiratory, thoracic and mediastinal disorders | 12,380 | Reliably estimable | 1.50 | 1.70 | 2.50 | 0.98 (0.84-1.14) | 0.774 | 0.30 (0.01-0.59) | 0.048 | Yes | No | No |
| steroid dependence | General disorders and administration site conditions | 628 | Reliably estimable | 1.27 | 0.49 | 26.65 | 42.61 (14.89-121.91) | <0.001 | 25.89 (20.48-31.31) | <0.001 | Yes | Yes | Yes |
| truncus arteriosus persistent | Congenital, familial and genetic disorders | 171 | Reliably estimable | 3.89 | 0.59 | 170.53 | 74.25 (8.73-631.38) | <0.001 | 167.05 (106.80-227.30) | <0.001 | Yes | Yes | Yes |
| urinary tract disorder | Renal and urinary disorders | 4,027 | Reliably estimable | 1.31 | 1.57 | 4.09 | 1.98 (1.55-2.54) | <0.001 | 2.21 (1.65-2.76) | <0.001 | Yes | Yes | Yes |
| vaginal discharge | Reproductive system and breast disorders | 9,327 | Reliably estimable | 0.61 | 0.67 | 1.66 | 4.07 (3.10-5.35) | <0.001 | 1.38 (1.10-1.67) | <0.001 | Yes | Yes | Yes |
| vaginal flatulence | Reproductive system and breast disorders | 243 | Reliably estimable | 1.94 | 9.66 | 88.71 | 4.73 (1.40-15.97) | 0.015 | 78.11 (53.76-102.46) | <0.001 | Yes | Yes | Yes |

**Note:** PT: Preferred Term; SOC: System Organ Class; OR: Odds Ratio; A: Aspirin exposure; B: Statin exposure; AB: Aspirin + statin co-exposure; IR: Interaction Ratio on the multiplicative scale; OR-based RERI: Relative excess due to interaction calculated from odds ratios on the additive odds scale; BH-FDR: Benjamini-Hochberg false discovery rate. IR > 1 with a 95% CI lower bound > 1 and BH-FDR < 0.05 indicates a positive multiplicative statistical interaction; OR-based RERI > 0 with a 95% CI lower bound > 0 and BH-FDR < 0.05 indicates a positive additive statistical interaction. These findings represent statistical pharmacovigilance interaction signals and do not establish causal pharmacological drug-drug interactions.

**Table S4.** Full adjusted statistical interaction results for all 42 candidate PTs.
