# Supplementary material for: Disproportionality analysis of adverse event reporting with aspirin, statins, and their co-exposure in FAERS, 2004–2025
Source: Front Med (Lausanne). 2026 Oct 1;13:1923188. doi: 10.3389/fmed.2026.1923188 (PMC13669111; doi:10.3389/fmed.2026.1923188)
Supplement: Supplementary file 5 [file Table5.docx]

| **PT** | **Primary SOC** | **Primary IR (95% CI)** | **Complete-case IR (95% CI)** | **IR direction consistent** | **Primary OR-based RERI (95% CI)** | **Complete-case OR-based RERI (95% CI)** | **RERI direction consistent** | **Complete-case estimable** | **Complete-case both-scale positive** | **Interpretation** |
| --- | --- | --- | --- | --- | --- | --- | --- | --- | --- | --- |
| adjustment disorder | Psychiatric disorders | 5.83 (3.73-9.11) | 8.18 (4.71-14.19) | Yes | 14.64 (12.03-17.25) | 19.15 (15.47-22.83) | Yes | Yes | Yes | Remained positive in complete-case analysis |
| affect lability | Psychiatric disorders | 3.81 (3.00-4.85) | 3.08 (2.32-4.09) | Yes | 1.75 (1.43-2.07) | 2.53 (1.97-3.08) | Yes | Yes | Yes | Remained positive in complete-case analysis |
| amaurosis fugax | Eye disorders | 2.16 (1.43-3.27) | 0.92 (0.51-1.65) | No | 11.98 (9.17-14.78) | 0.17 (-0.95-1.29) | Yes | Yes | No | Direction not fully consistent |
| blood calcium decreased | Investigations | 1.22 (1.05-1.41) | 1.21 (1.01-1.44) | Yes | 0.83 (0.53-1.13) | 0.71 (0.37-1.05) | Yes | Yes | No | Directionally consistent but no longer jointly significant |
| burns second degree | Injury, poisoning and procedural complications | 5.46 (3.48-8.55) | 6.35 (3.53-11.42) | Yes | 5.08 (3.95-6.21) | 4.69 (3.41-5.96) | Yes | Yes | Yes | Remained positive in complete-case analysis |
| carotid artery thrombosis | Nervous system disorders | 2.56 (1.62-4.03) | 2.38 (1.44-3.92) | Yes | 19.87 (15.40-24.35) | 24.85 (18.80-30.90) | Yes | Yes | Yes | Remained positive in complete-case analysis |
| catarrh | Respiratory, thoracic and mediastinal disorders | 9.91 (4.42-22.24) | 11.10 (3.88-31.75) | Yes | 12.14 (9.33-14.95) | 9.02 (6.35-11.68) | Yes | Yes | Yes | Remained positive in complete-case analysis |
| colitis ulcerative | Gastrointestinal disorders | 1.96 (1.72-2.22) | 1.93 (1.66-2.25) | Yes | 0.64 (0.51-0.76) | 0.65 (0.50-0.80) | Yes | Yes | Yes | Remained positive in complete-case analysis |
| congenital cardiovascular anomaly | Congenital, familial and genetic disorders | 282.46 (84.58-943.28) | NA | NA | 10.79 (8.00-13.57) | NA | NA | No | NA | Not estimable in complete-case analysis |
| creatinine renal clearance decreased | Investigations | 1.32 (1.08-1.62) | 1.16 (0.92-1.47) | Yes | 1.61 (1.09-2.13) | 1.26 (0.68-1.85) | Yes | Yes | No | Directionally consistent but no longer jointly significant |
| female genital tract fistula | Reproductive system and breast disorders | 3.46 (2.28-5.26) | 4.07 (2.48-6.69) | Yes | 5.33 (3.99-6.68) | 6.03 (4.40-7.65) | Yes | Yes | Yes | Remained positive in complete-case analysis |
| frequent bowel movements | Gastrointestinal disorders | 1.33 (1.17-1.51) | 1.28 (1.10-1.50) | Yes | 0.48 (0.30-0.66) | 0.40 (0.19-0.61) | Yes | Yes | Yes | Remained positive in complete-case analysis |
| gingival pain | Gastrointestinal disorders | 1.27 (1.04-1.53) | 1.30 (1.03-1.64) | Yes | 0.64 (0.31-0.98) | 0.58 (0.21-0.95) | Yes | Yes | Yes | Remained positive in complete-case analysis |
| impaired quality of life | Social circumstances | 1.55 (1.28-1.86) | 0.95 (0.77-1.18) | No | 0.63 (0.32-0.95) | 0.16 (-0.25-0.58) | Yes | Yes | No | Direction not fully consistent |
| malignant melanoma in situ | Neoplasms benign, malignant and unspecified (incl cysts and polyps) | 1.91 (1.31-2.78) | 1.24 (0.82-1.86) | Yes | 2.58 (1.59-3.56) | 1.52 (0.41-2.63) | Yes | Yes | No | Directionally consistent but no longer jointly significant |
| muscle spasticity | Nervous system disorders | 3.20 (2.62-3.90) | 3.65 (2.89-4.61) | Yes | 1.56 (1.30-1.82) | 2.09 (1.73-2.46) | Yes | Yes | Yes | Remained positive in complete-case analysis |
| nasal oedema | Respiratory, thoracic and mediastinal disorders | 3.10 (2.09-4.60) | 3.97 (2.45-6.46) | Yes | 4.33 (3.20-5.46) | 4.94 (3.58-6.29) | Yes | Yes | Yes | Remained positive in complete-case analysis |
| needle fatigue | Injury, poisoning and procedural complications | 50.42 (10.44-243.47) | 55.17 (6.01-506.82) | Yes | 17.28 (12.27-22.28) | 45.20 (29.02-61.39) | Yes | Yes | Yes | Remained positive in complete-case analysis |
| neurologic neglect syndrome | Nervous system disorders | 2.39 (1.35-4.21) | 2.15 (1.18-3.93) | Yes | 54.69 (41.22-68.16) | 55.39 (40.46-70.31) | Yes | Yes | Yes | Remained positive in complete-case analysis |
| ocular discomfort | Eye disorders | 1.39 (1.13-1.69) | 0.77 (0.57-1.04) | No | 0.88 (0.51-1.24) | -0.32 (-0.65-0.00) | No | Yes | No | Direction not fully consistent |
| personality disorder | Psychiatric disorders | 3.25 (2.34-4.51) | 5.51 (3.58-8.49) | Yes | 4.75 (3.76-5.75) | 7.12 (5.71-8.52) | Yes | Yes | Yes | Remained positive in complete-case analysis |
| proctitis | Gastrointestinal disorders | 2.02 (1.48-2.77) | 1.74 (1.21-2.50) | Yes | 2.31 (1.58-3.04) | 2.13 (1.27-2.99) | Yes | Yes | Yes | Remained positive in complete-case analysis |
| radiculopathy | Nervous system disorders | 1.44 (1.10-1.87) | 1.47 (1.07-2.01) | Yes | 4.21 (3.13-5.29) | 3.93 (2.72-5.13) | Yes | Yes | Yes | Remained positive in complete-case analysis |
| relapsing-remitting multiple sclerosis | Nervous system disorders | 7.84 (3.72-16.51) | 16.52 (6.27-43.55) | Yes | 6.57 (4.52-8.62) | 9.39 (6.57-12.22) | Yes | Yes | Yes | Remained positive in complete-case analysis |
| sedation | Nervous system disorders | 2.29 (1.97-2.65) | 2.13 (1.79-2.53) | Yes | 0.79 (0.65-0.94) | 0.73 (0.57-0.90) | Yes | Yes | Yes | Remained positive in complete-case analysis |
| sedation complication | Injury, poisoning and procedural complications | 3.76 (2.52-5.60) | 3.13 (2.02-4.83) | Yes | 6.59 (5.28-7.90) | 5.24 (3.93-6.56) | Yes | Yes | Yes | Remained positive in complete-case analysis |
| steroid dependence | General disorders and administration site conditions | 42.61 (14.89-121.91) | 22.76 (7.43-69.71) | Yes | 25.89 (20.48-31.31) | 21.75 (15.68-27.82) | Yes | Yes | Yes | Remained positive in complete-case analysis |
| truncus arteriosus persistent | Congenital, familial and genetic disorders | 74.25 (8.73-631.38) | 3.19 (0.25-40.00) | Yes | 167.05 (106.80-227.30) | 784.20 (-61.47-1629.87) | Yes | Yes | No | Directionally consistent but no longer jointly significant |
| urinary tract disorder | Renal and urinary disorders | 1.98 (1.55-2.54) | 1.28 (0.92-1.80) | Yes | 2.21 (1.65-2.76) | 0.46 (-0.03-0.95) | Yes | Yes | No | Directionally consistent but no longer jointly significant |
| vaginal discharge | Reproductive system and breast disorders | 4.07 (3.10-5.35) | 3.59 (2.60-4.95) | Yes | 1.38 (1.10-1.67) | 1.45 (1.06-1.83) | Yes | Yes | Yes | Remained positive in complete-case analysis |
| vaginal flatulence | Reproductive system and breast disorders | 4.73 (1.40-15.97) | 3.75 (0.84-16.71) | Yes | 78.11 (53.76-102.46) | 106.84 (62.39-151.29) | Yes | Yes | No | Directionally consistent but no longer jointly significant |

**Note:** PT: Preferred Term; SOC: System Organ Class; IR: Interaction Ratio on the multiplicative scale; OR-based RERI: Relative excess due to interaction calculated from odds ratios on the additive odds scale; Primary: primary adjusted statistical interaction analysis; Complete-case: sensitivity analysis restricted to reports with complete covariate information; Direction consistent: the interaction estimate remained in the same direction in the primary and complete-case analyses. NA: Not available. Loss of statistical significance in the complete-case analysis does not necessarily indicate reversal of the association because the complete-case dataset is substantially smaller.

**Table S5.** Complete-case sensitivity analysis of the 31 primary both-scale-positive PTs.
