# Supplementary material for: Disproportionality analysis of adverse event reporting with aspirin, statins, and their co-exposure in FAERS, 2004–2025
Source: Front Med (Lausanne). 2026 Oct 1;13:1923188. doi: 10.3389/fmed.2026.1923188 (PMC13669111; doi:10.3389/fmed.2026.1923188)
Supplement: Supplementary file 6 [file Table6.docx]

**Table S6.**  Complete-case sensitivity-analysis retention and missing-data summary.

| **Item** | **Value** | **Unit** |
| --- | --- | --- |
| **Complete-case retention** |  |  |
| Reports in primary analysis | 18,932,599 | reports |
| Complete-case reports | 10,190,569 | reports |
| Reports excluded from complete-case analysis | 8,742,030 | reports |
| Complete-case retention | 53.83 | % |
| Complete-case exclusion | 46.17 | % |
| **Missingness of adjustment covariates** | **Missing/unknown, (n,%)** | **Primary-analysis handling** |
| Age | 8,013,417 (42.33) | Unknown category |
| Sex | 2,295,134 (12.12) | Unknown category |
| Reporter type | 823,543 (4.35) | Unknown category |
| Reporting year | 0 (0.00) | No missing reports |

**Note:** Unknown category: reports with missing covariate values retained as an explicit Unknown category in the primary analysis; Complete-case analysis: sensitivity analysis restricted to reports with complete covariate information; Retention: percentage of primary-analysis reports retained in the complete-case dataset; Exclusion: percentage of primary-analysis reports removed from the complete-case dataset.
